# GPRC5B preserves a mature **β** cell state in obesity by controlling MafA expression

**DOI:** 10.1172/jci.insight.194115

**Published:** 2025-09-04

**Authors:** Tianpeng Wang, Remy Bonnavion, Janett Piesker, Stefan Günther, Nina Wettschureck

**Affiliations:** 1 Department of Pharmacology, Max Planck Institute for Heart and Lung Research, Bad Nauheim, Germany.; 2 Imaging Platform, Max Planck Institute for Heart and Lung Research, Bad Nauheim, Germany.; 3 Bioinformatics and Deep Sequencing Platform, Max Planck Institute for Heart and Lung Research, Bad Nauheim, Germany.; 4 Medical Faculty, J.W. Goethe University Frankfurt, Germany.

**Keywords:** Endocrinology, Metabolism, Beta cells, G protein-coupled receptors, Obesity

## Abstract

In vitro studies have implicated orphan receptor GPRC5B in β cell survival, proliferation, and insulin secretion, but its relevance for glucose homeostasis in vivo is largely unknown. Using tamoxifen-inducible, β cell–specific GPRC5B-KO mice (Ins-G5b–KOs), we show here that loss of GPRC5B does not affect β cell function in the lean state but results in strongly reduced insulin secretion and disturbed glucose tolerance in mice subjected to high-fat diet for 16 weeks. Flow cytometry and single-cell expression analyses in islets from obese mice show a reduced β cell abundance and a less mature β cell phenotype in Ins-G5b–KOs. Expression of β cell–specific transcription factor MafA is reduced both on the RNA and protein level, as are transcripts of MafA target genes. Mechanistically, we show that phosphorylation of cAMP response element-binding protein (CREB), a major regulator of MafA expression, is reduced in islets of obese Ins-G5b–KOs, and we show that this phenotype precedes the downregulation of MafA and MafA target genes. Taken together, GPRC5B helps to maintain mature β cell function in obesity through cAMP/CREB-dependent regulation of MafA expression.

## Abstract 2

Orphan receptor GPRC5B maintains mature β-cell function in obesity via cAMP/CREB-dependent MafA regulation; β-cell-specific GPRC5B-knockout mice have reduced insulin secretion and develop a prediabetic condition.

## Introduction

The ability of pancreatic β cells to secrete adequate amounts of insulin is crucial for maintaining normoglycemia. When insulin secretion is compromised, it can lead to diabetes mellitus, characterized by hyperglycemia, dyslipidemia, and subsequent long-term damage to tissues (1). Insulin release from β cells is primarily regulated by blood glucose levels. Elevated glucose concentrations enhance intracellular glucose metabolism, leading to an increase in adenosine triphosphate (ATP). This triggers the closure of ATP-sensitive potassium channels, which in turn opens voltage-operated calcium channels, resulting in the calcium-driven exocytosis of insulin-containing vesicles (2, 3).

While the ATP-dependent pathway is the primary regulator of insulin release, several other factors enhance insulin secretion in response to glucose. Gastrointestinal hormones like glucagon-like peptide-1 (GLP-1), glucose-dependent insulinotropic polypeptide, and pituitary adenylate cyclase-activating polypeptide boost insulin secretion by engaging G-protein-coupled receptors (GPCRs). These receptors signal through the G_s_ family of heterotrimeric G-proteins (4). The enhancement of glucose-induced insulin release by G_s_ involves the activation of adenylyl cyclase, which leads to the subsequent cAMP-dependent activation of protein kinase A and Epac2 (4). These prosecretory effects are counteracted by activation of G_i/o_-coupled GPCRs such as the α_2_-adrenergic receptor, the somatostatin receptors SST_2_ and SST_5_, or the acetate receptors FFA2 and FFA3, since activated G_i/o_ family G-proteins inhibit adenylyl cyclase (4). Other GPCRs with known function in the regulation of insulin secretion are the G_q/11_-coupled muscarinic receptor subtype M_3_ or the free fatty acid receptors FFA1 and FFA4 (4). In addition to their role in insulin secretion, GPCRs may help to maintain adequate β cell function under stressful metabolic conditions — for example, by regulating the expression of key transcription factors controlling β cell maturation such as MafA and PDX1 (5).

Interestingly, pancreatic β cells express many more GPCRs than the above-mentioned receptors. mRNA-Seq detected as many as 229 GPCRs in murine β cells (6), though some of them at very low levels. Among those with more robust expression (>1 RPKM), numerous GPCRs are still “orphan”—i.e., the endogenous ligand is unknown. One example is G Protein-Coupled Receptor Class C Group 5 Member B (GPRC5B). GPRC5B is expressed in murine and human β cells (6, 7) but also in other islet cells (6) and extrapancreatic cells such as neurons, fibroblasts, or smooth muscle cells (8, 9). Constitutive deletion of the gene encoding GPRC5B, *Gprc5b*, results in mice in altered neurogenesis and behavioral abnormalities (10–12); in addition, GPRC5B-KOs are protected from diet-induced obesity (13). We have recently studied the function of GPRC5B in vascular smooth muscle cells and macrophages, and we found that it regulates both cell types by modulating the intracellular trafficking of G_s_-coupled GPCRs such as the prostacyclin receptor or the prostaglandin E2 receptor EP2 (14, 15).

The role of GPRC5B in β cells is not well defined. It was previously shown that knockdown of *Gprc5b* in cultured murine islets resulted in enhanced basal and glucose-stimulated insulin secretion (GSIS) (16), but which islet cell type was responsible for these changes is not clear. In global GPRC5B-KO mice, circulating insulin levels were reduced especially in diet-induced obesity (13, 17), but since KOs also showed a lean phenotype with reduced insulin resistance and lower obesity-associated inflammation (13), these changes might have been secondary. Finally, in vitro studies in MIN6 insulinoma cells suggested a role of GPRC5B in β cell proliferation and apoptosis (18). However, none of these studies analyzed insulin secretion in a β cell–specific manner, nor did they address the role of GPRC5B in β cell function in vivo. We have therefore generated tamoxifen-inducible, β cell–specific KO mice for *Gprc5b* and studied the role of this receptor in insulin secretion. We found that loss of GPRC5B did not affect insulin secretion in lean mice but resulted in lowered activity of the cAMP/CREB signaling cascade and reduced expression of transcription factor MafA. In the obese state, reduced MafA expression was associated with a less mature β cell phenotype in KOs, resulting in reduced insulin secretion and reduced glucose tolerance in vivo.

## Results

### Generation and characterization of tamoxifen-inducible, β cell–specific GPRC5B-KO mice.

**Figure 1:**
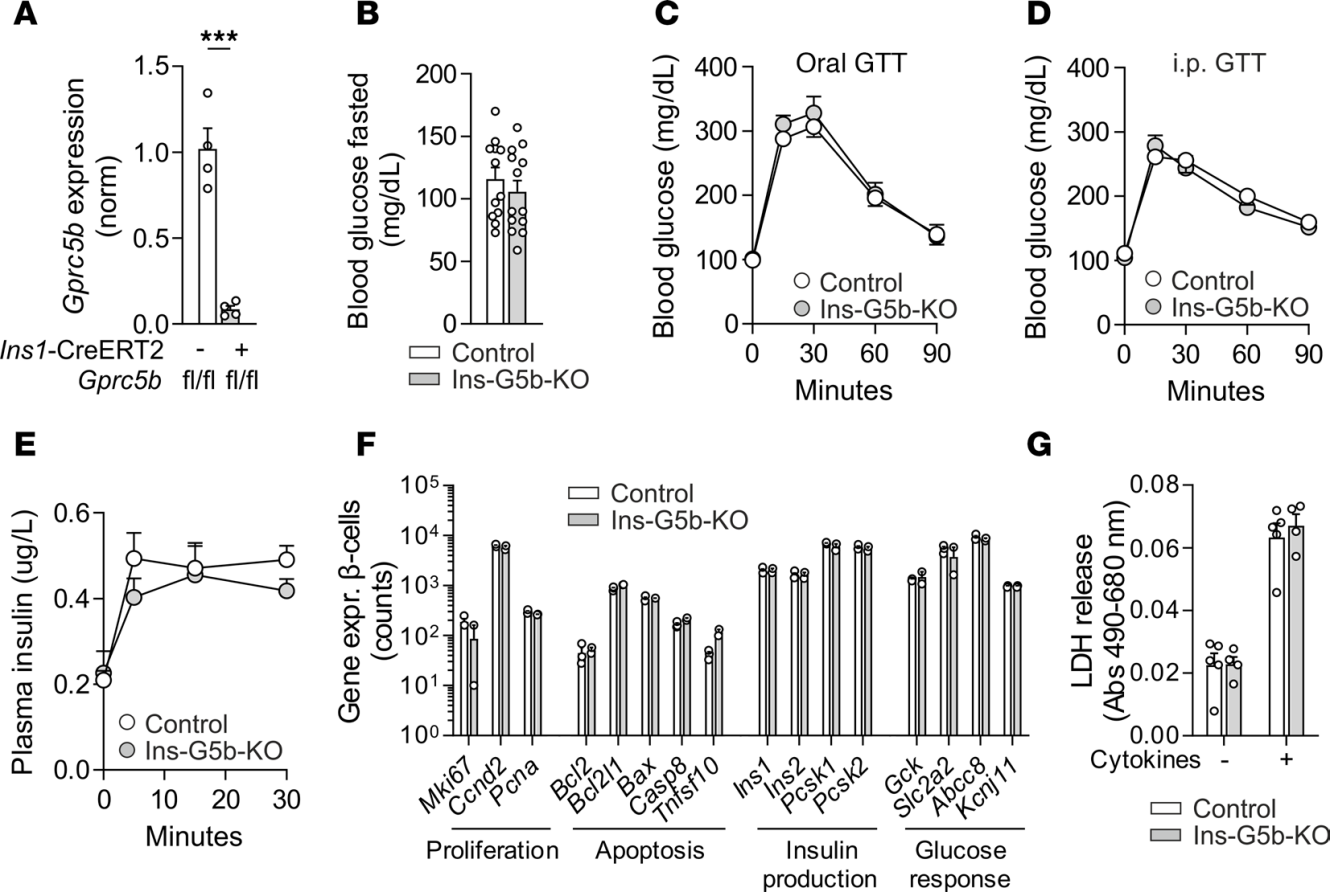
General characterization of tamoxifen-inducible, β cell–specific GPRC5B-KO mice (Ins-G5b–KOs). (**A**) *Gprc5b* KO efficiency was determined by qPCR in pancreatic β cells from control and Ins-G5b–KOs that had been bred to a Cre-dependent reporter line to allow β cell isolation by flow cytometry (*n* = 4/4 mice). (**B**) Blood glucose levels in control and Ins-G5b–KO mice after 6 hours of fasting (*n* = 12/13 mice). (**C**–**E**) Blood glucose levels (**C** and **D**) and insulin levels (**E**) in control and Ins-G5b–KOs after oral (**C**) or i.p. (**D** and **E**) application of glucose (**C**, *n* = 12/12 mice, **D**, *n* = 12/12 mice, **E**, *n* = 12/12 mice). (**F**) mRNA-Seq in flow cytometry–isolated β cells harvested from control and Ins-G5b–KO mice that had been bred to a Cre-dependent reporter line to allow β cell isolation by flow cytometry (*n* = 3/2 mice). The *y* axis represents DESeq2 normalized counts. (**G**) Islet viability was determined by lactate dehydrogenase (LDH) cytotoxicity kit after 24 hours of incubation in the absence or presence of a cytokine cocktail (100 ng/mL IL-1β, 125 ng/mL TNF-α, and 125 ng/mL INF-γ) in culture media. Data are presented as absorbance (Abs) values (*n* = 5/4 mice). Data are mean ± SEM; comparisons between control and KO samples were performed using unpaired Student’s *t* test (**A** and **B**; 2-way repeated measures ANOVA with Šidák’s multiple-comparison test (**C**–**E**); multiple unpaired Student’s *t* test with 2-stage linear step-up procedure of Benjamini, Krieger, and Yekutieli (**F**); or 2-way ANOVA with Šidák’s multiple-comparison test (**G**). ***P ≤ 0.001.

To generate tamoxifen-inducible, β cell–specific GPRC5B-deficient mice, we crossed mice carrying a floxed *Gprc5b* allele (15) with the *Ins1*-CreERT2 line (19), resulting in *Ins1*-CreERT2^+^ *Gprc5b^fl/fl^* mice (Ins-G5b–KOs) and their respective *Gprc5b^fl/fl^* littermate controls. To enable isolation of pancreatic β cells by FACS, some of these mice were additionally bred to the Cre-dependent fluorescent reporter construct mTmG (Rosa26flox-mT-stop-flox-mG) (20). Three weeks after tamoxifen treatment, flow cytometry–isolated pancreatic β cells from Ins-G5b–KOs showed significantly reduced *Gprc5b* expression compared with controls (Figure 1A), but this was not associated with changes in fasting blood glucose levels or altered responses to oral or i.p. glucose-tolerance test (GTT) (Figure 1, B–D, Supplemental Figure 1, A and B; supplemental material available online with this article; https://doi.org/10.1172/jci.insight.194115DS1). Furthermore, plasma insulin levels did not reveal clear differences after i.p. glucose challenge (Figure 1E). mRNA-Seq of β cells sorted from control and KO islets did not show differences in expression of genes related to proliferation, apoptosis, insulin production, glucose responses, or GPCRs implicated in modulation of insulin secretion (Figure 1F and Supplemental Figure 1C). Furthermore, we did not observe differences in the viability of cultured islets in the basal state or after exposure to cytokines known to induce cytotoxic effects (Figure 1G), and we also did not observe differences in β cell proliferation or apoptosis (Supplemental Figure 1, D and E). Taken together, β cell–specific inactivation of GPRC5B does not have obvious consequences for glucose homeostasis or islet survival in lean mice.

### Impaired glucose tolerance and insulin secretion in obese Ins-G5b–KOs.

**Figure 2:**
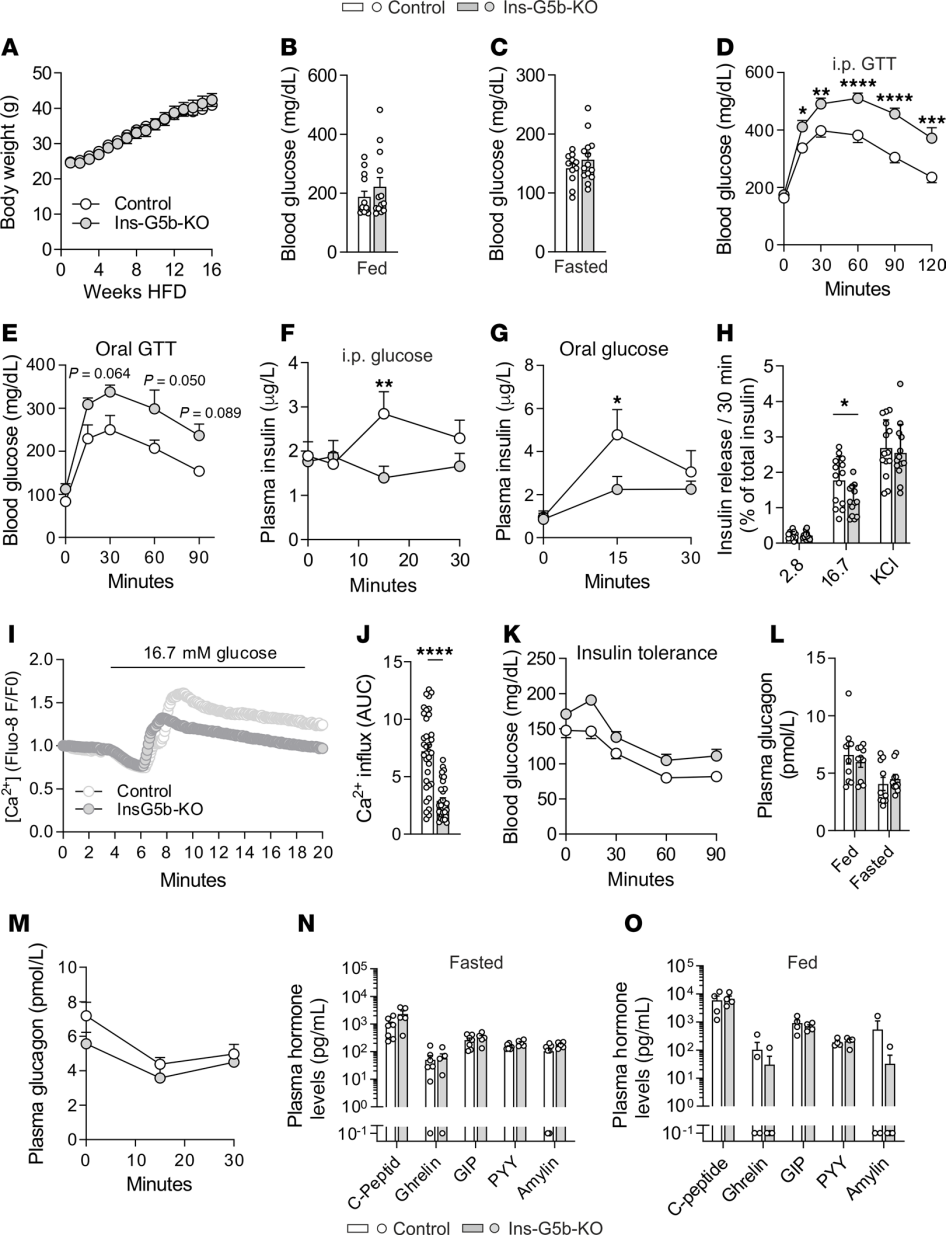
Metabolic profiling of Ins-G5b–KO mice after 16 weeks of HFD feeding. (**A**) Body weight gain in control and Ins-G5b–KOs (*n* = 21/21 mice) during 16 weeks of HFD feeding. (**B** and **C**) Blood glucose levels in the fed (**B**, *n* = 13/13 mice) and 12-hour–fasted state (**C**, *n* = 11/14 mice). (**D** and **E**) Blood glucose levels after i.p. (**D**) or oral (**E**) administration of glucose (1.5 g/kg body weight) (**D**, *n* = 19/21 mice, **E**, *n* = 5/5 mice). (**F** and **G**) Plasma insulin levels after i.p. (**F**) or oral (**G**) administration of glucose (1.5 g/kg body weight) (**F**, *n* = 24/24 mice, **G**, *n* = 4/5 mice). (**H**) Insulin secretion from isolated islets of obese control mice and Ins-G5b–KOs in response to 2.8 mM glucose (2.8), 16.7 mM glucose (16.7), or 30 mM KCl (*n* = 14/13 mice). (**I** and **J**) Glucose-induced calcium mobilization in Fluo-8–loaded islets: **I**, original traces, **J**, quantification of areas under the curve (AUC) (*n* = 35/32 islets from 3/4 mice). (**K**) Blood glucose levels after i.p. application of 0.75 U/kg body weight insulin (insulin tolerance test, *n* = 12/12 mice). (**L**) Plasma glucagon levels were determined in the fed and fasted state by ELISA (*n* = 10/10 mice). (**M**) Plasma glucagon levels after i.p. application of 1.5 g/kg glucose (*n* = 10/8 mice). (**N** and **O**) Plasma levels of gastrointestinal hormones and mediators implicated in glucose homeostasis in the fasted (**N**, *n* = 7/5 mice) and fed state (**O**, *n* = 4/4 mice) by MILLIPLEX Assay. Samples below detection limit were set to 0.1 pg/mL for visualization. Data are mean ± SEM; comparisons between control and KO samples were performed using unpaired Student’s *t* test (**B**, **C**, and **J**), 2-way repeated measures ANOVA with Šidák’s multiple-comparison test (**A**, **D**–**H**, and **K**–**M**), and multiple unpaired Student’s *t* test with 2-stage linear step-up procedure of Benjamini, Krieger ,and Yekutieli (**N** and **O**). *P ≤ 0.05, **P ≤ 0.01, ***P ≤ 0.001, ****P ≤ 0.0001.

Previous studies (16) suggested that *Gprc5b* expression is upregulated during type 2 diabetes, which led us to investigate the role of GPRC5B in mice with diet-induced obesity, a model that is associated with development of insulin resistance and type 2 diabetes (21). When fed for 16 weeks with high-fat diet (HFD), Ins-G5b–KOs showed the same increase in body weight as control mice (Figure 2A), and fed and fasted blood glucose levels did not differ significantly (Figure 2, B and C). A more detailed analysis of glucose homeostasis, however, revealed clearly increased and prolonged hyperglycemia in both i.p. and oral GTT in Ins-G5b–KOs (Figure 2, D and E, and Supplemental Figure 2, A and B). In line with a disturbed glucose tolerance, plasma insulin levels of obese Ins-G5b–KOs were strongly reduced after glucose challenge (Figure 2, F and G). We next examined insulin secretion in vitro using isolated islets. Islets from obese Ins-G5b–KOs exhibited significantly reduced glucose-induced insulin secretion compared with control islets, whereas basal and potassium chloride–induced (KCl-induced) insulin secretion did not differ (Figure 2H). Consistently, glucose-induced calcium influx was reduced in GPRC5B-deficient islets (Figure 2, I and J). Apart from this impairment of glucose-induced insulin secretion, we did not observe abnormalities in peripheral insulin sensitivity by i.p. insulin-tolerance test (ITT) (Figure 2K and Supplemental Figure 2C), plasma glucagon levels in the fed or fasted state (Figure 2L), glucose-induced suppression of glucagon levels (Figure 2M), or plasma levels of other pancreatic and gastrointestinal mediators related to glucose homeostasis (Figure 2, N and O). Also histological analysis of adipose tissue did not show significant differences between the genotypes (Supplemental Figure 2, D–F), and HFD did not induce significant changes in *Gprc5b* expression (Supplemental Figure 2G). Taken together, Ins-G5b–KOs subjected to diet-induced obesity show reduced glucose-induced insulin secretion in vitro and in vivo, and glucose tolerance is impaired.

### Total insulin content and β cell proportion is reduced in obese Ins-G5b–KOs.

**Figure 3:**
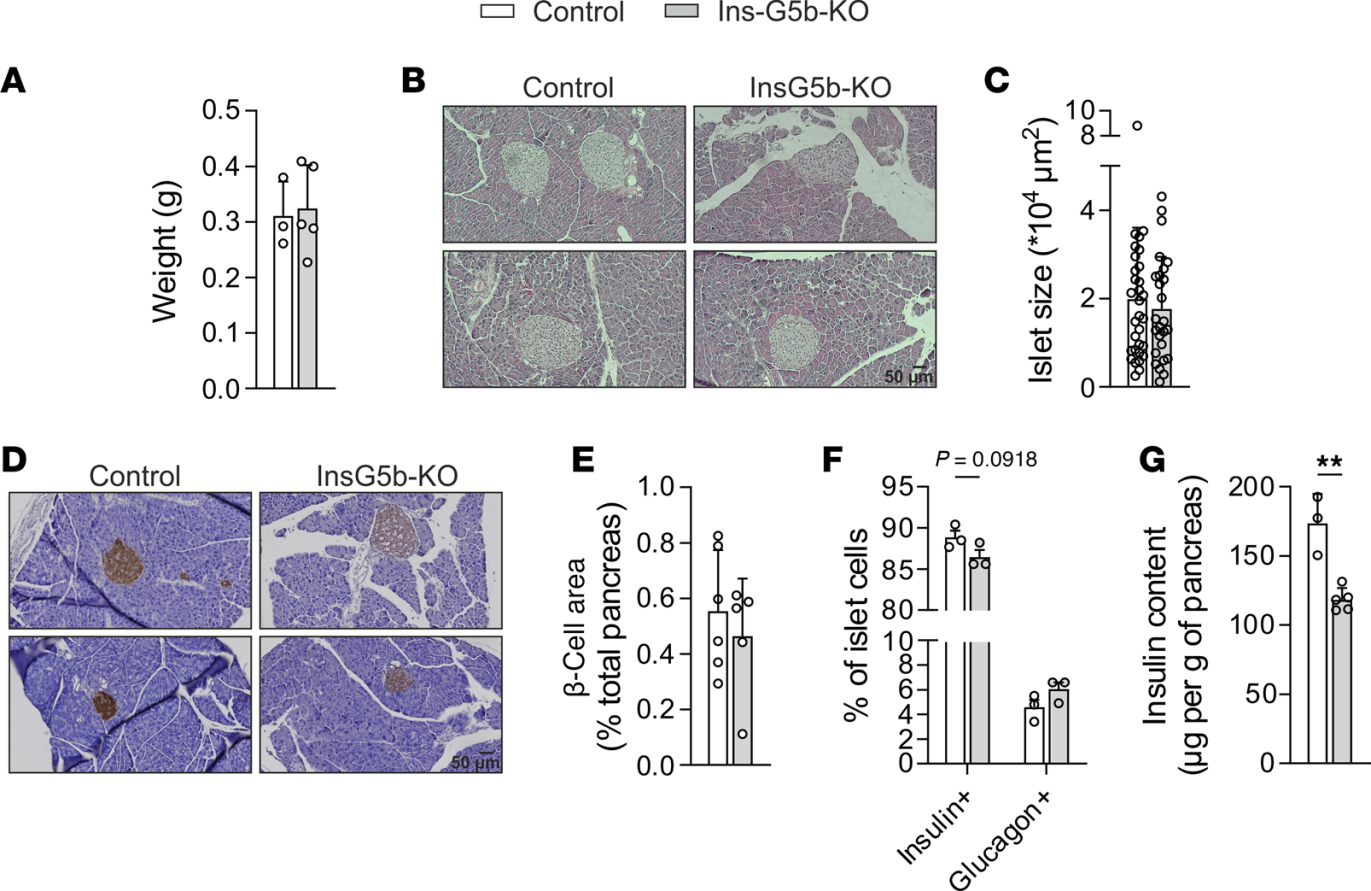
Reduced insulin content in obese Ins-G5b–KOs. (**A**) Pancreas weight in control and Ins-G5b–KO mice fed for 16 weeks with HFD (*n* = 3/5 mice). (**B** and **C**) Histological analysis of islet morphology in pancreata of HFD-fed control and Ins-G5b–KOs. Exemplary microphotographs (**B**) and quantification of islet size (**C**) (*n* = 31/26 individual islets from 6/5 mice). (**D** and **E**) Insulin labeling in pancreata from HFD-fed mice. Exemplary microphotographs (**D**) and quantification of β cell area ratio per total pancreas area (**E**) (*n* = 6/5 mice). (**F**) Flow cytometric analysis of the proportion of insulin^+^ β cells and glucagon^+^ α cells in islets of obese control and Ins-G5b–KO mice (*n* = 3/3 mice). (**G**) Insulin content in pancreata was determined by ELISA in obese control and Ins-G5b–KO mice (*n* = 3/5 mice). Data are mean ± SEM; comparisons between control and KO samples were performed using unpaired Student’s *t* test (**A**, **C**, **E**, and **G**) and 2-way repeated measures ANOVA with Šidák’s multiple-comparison test (**F**). *P ≤ 0.05, **P ≤ 0.01, ***P ≤ 0.001. Scale bars: 50 μm.

To understand how loss of GPRC5B contributes to β cell function under conditions of HFD, we determined pancreas weight and islet size in pancreas sections from obese mice but did not observe significant differences between the genotypes (Figure 3, A–C). Insulin immunostaining revealed a mild and nonsignificant reduction of β cell area (Figure 3, D and E). In addition, flow cytometric analysis of the proportion of β and α cells within islets of HFD-fed mice showed that the percentage of insulin^+^ β cells was slightly reduced, whereas glucagon^+^ α cells were relatively increased, though also these changes did not reach statistical significance (Figure 3F). In line with this, Ins-G5b–KO pancreata showed lower total insulin content (Figure 3G). Further analyses in β cells from obese Ins-G5b–KOs did not show differences with respect to mitochondrial number and morphology, reactive oxygen species (ROS) production, or ATP/ADP ratio compared with control mice (Supplemental Figure 3).

### GPRC5B deficiency reduces expression of MafA and MafA-dependent genes in the obese state.

**Figure 4:**
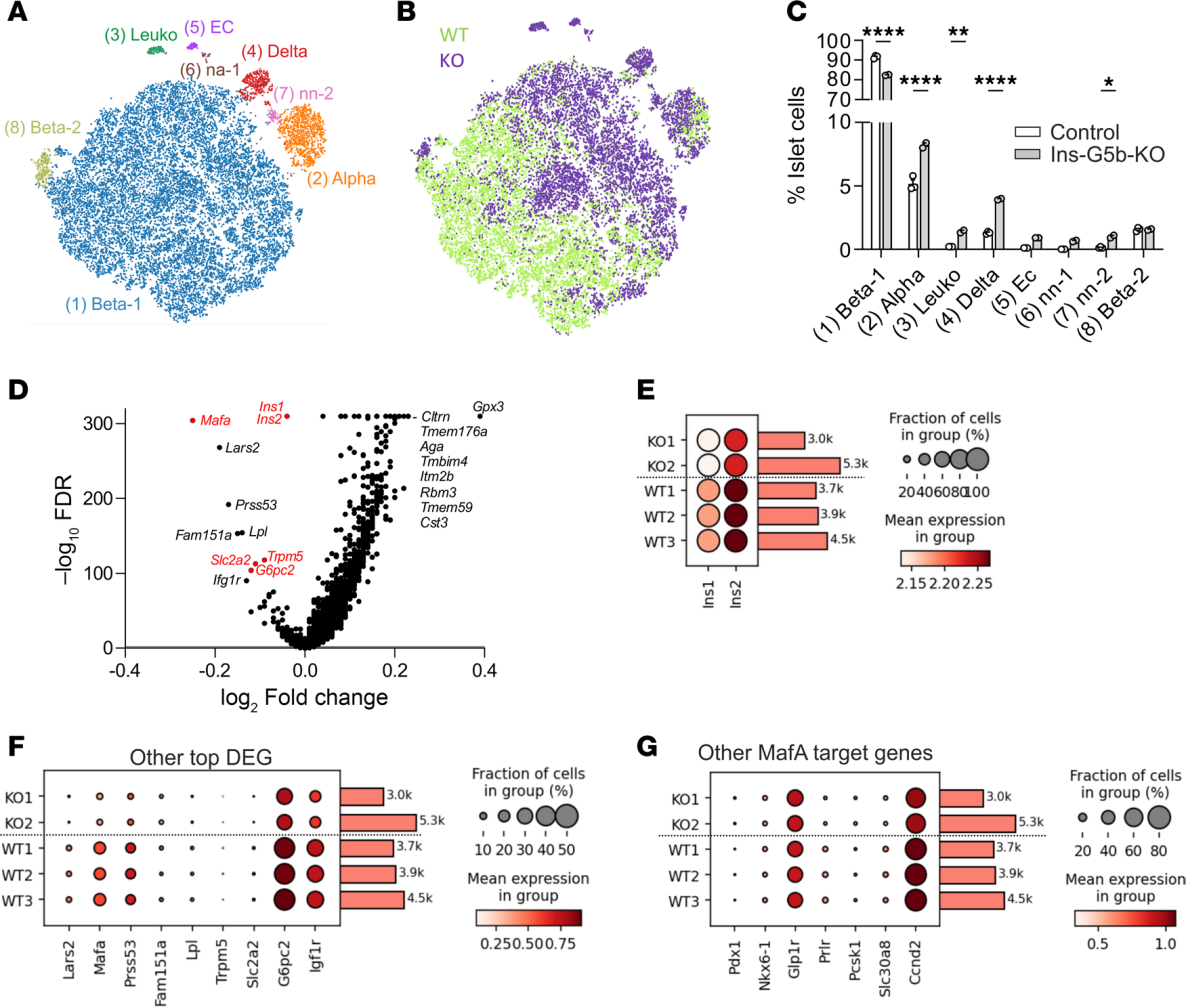
Single-cell transcriptome characterization of pancreatic islets from obese Ins-G5b–KOs. (**A**) t-SNE plot showing clustering of islet cells harvested from 3 control mice (WT1–WT3, *n* = 12,928 cells) and 2 Ins-G5b–KO mice (KO1 and KO2, *n* = 9,889) fed for 16 weeks with HFD. (**B** and **C**) Contribution of control (WT) and KO cells to the different clusters: t-SNE plot (**B**) and relative cluster size in individual mice (**C**). (**D**) Volcano plot showing genes that there are differentially expressed in β cells from obese Ins-G5b–KOs versus obese control mice. Genes with a well-established role in β cell biology are shown in red. (**E** and **F**) Dot plots showing expression frequency (%) and strength of top 10 downregulated genes in β cell clusters 1 and 8 from control (WT) and Ins-G5b–KOs (KO). (**G**) Dot plots showing expression levels of other MafA target genes within the β cell clusters. Data are mean ± SEM; comparisons between control and KO samples were performed using 2-way repeated measures ANOVA with Šidák’s multiple-comparison test (**C**). EC, endothelial cells; Leuko, leukocytes; na-1/na-2, not yet annotated clusters 1 and 2. *P ≤ 0.05, **P ≤ 0.01, ****P ≤ 0.0001.

To study cellular composition and differentiation state of individual islet cell populations in more detail, we performed single-cell expression analyses in 16-week HFD-fed control and KO mice (Figure 4). Eight cell clusters were identified within pancreatic islets, among them 2 β cell clusters (beta-1 and beta-2), α cells, δ cells, leukocytes, endothelial cells, and two not yet annotated clusters (na-1, na-2) (Figure 4A). In line with our flow cytometric analyses, the percentage of cells in cluster 1, which makes up the vast majority of β cells, was reduced in KO mice, whereas all other populations were relatively increased (Figure 4, B and C).

To better understand the mechanism underlying altered β cell function in obese Ins-G5b–KOs, we analyzed differential gene expression. While gene set enrichment analysis did not reveal changes in metabolism-related pathways in α and δ cell clusters, numerous genes were differentially expressed in GPRC5B-deficient β cells (Figure 4D and Supplemental Figure 4). KEGG pathway enrichment analysis for upregulated genes identified pathways that were not specifically related to β cell function — e.g., various neurodegenerative disorders (Supplemental Figure 4, A and B). Additionally, we observed an enrichment of genes involved in oxidative phosphorylation, which might be of relevance to β cell function. However, we did not find direct evidence of altered ROS production in GPRC5B-deficient β cells in a functional assay (Supplemental Figure 3). In the downregulated genes, however, KEGG results indicated a strong correlation between these genes and diabetes mellitus (Supplemental Figure 4, C and D), prominently among them *Ins1* and *Ins2*, the 2 insulin encoding genes (Figure 4, D and E). Also, MafA was among the top downregulated genes, a transcription factor crucial for β cell maturation and insulin expression (22, 23) (Figure 4, D and F). Notably, several of the other top downregulated genes are not only key players in glucose-induced insulin secretion, but they are also directly or indirectly regulated by MafA (Figure 4, D and F) (22): *Slc2a2* encodes the predominant β cell glucose transporter GLUT-2(23); *G6pc2* encodes the catalytic subunit 2 of glucose-6-phosphatase, a key player in the conversion of glucose to ATP (23); and *Trpm5* encodes transient receptor potential cation channel subfamily M member, an important regulator of calcium influx in β cells (22) (Figure 4, D and F). Other differentially expressed genes are not known as MafA targets but have been implicated in β cell function, such as *Igf1r*, a regulator of β cell senescence and function (24) (Figure 4, D and F). However, other genes, such as *Lars2*, *Prss53*, *Fam151a*, or *Lpl*, have so far not been linked to β cell biology. Further analyses revealed that other MafA target genes (22), such as *Ccnd2*, *Prlr*, and *Slc30a8*, were reduced, whereas MafA target genes *Nkx6-1*, *Glp1r*, or *Pcsk1* were not significantly changed in GPRC5B-deficient β cells (Figure 4G). These findings demonstrate that GPRC5B deficiency in β cells results after exposure to HFD in reduced β cell abundance and impaired β cell phenotype maintenance, including reduced expression of key regulators of β cell function such as *Mafa*, *G6pc2*, and *Slc2a2* as well as insulin itself.

### GPRC5B regulates the mature β cell phenotype through the cAMP/CREB/MafA pathway.

**Figure 5:**
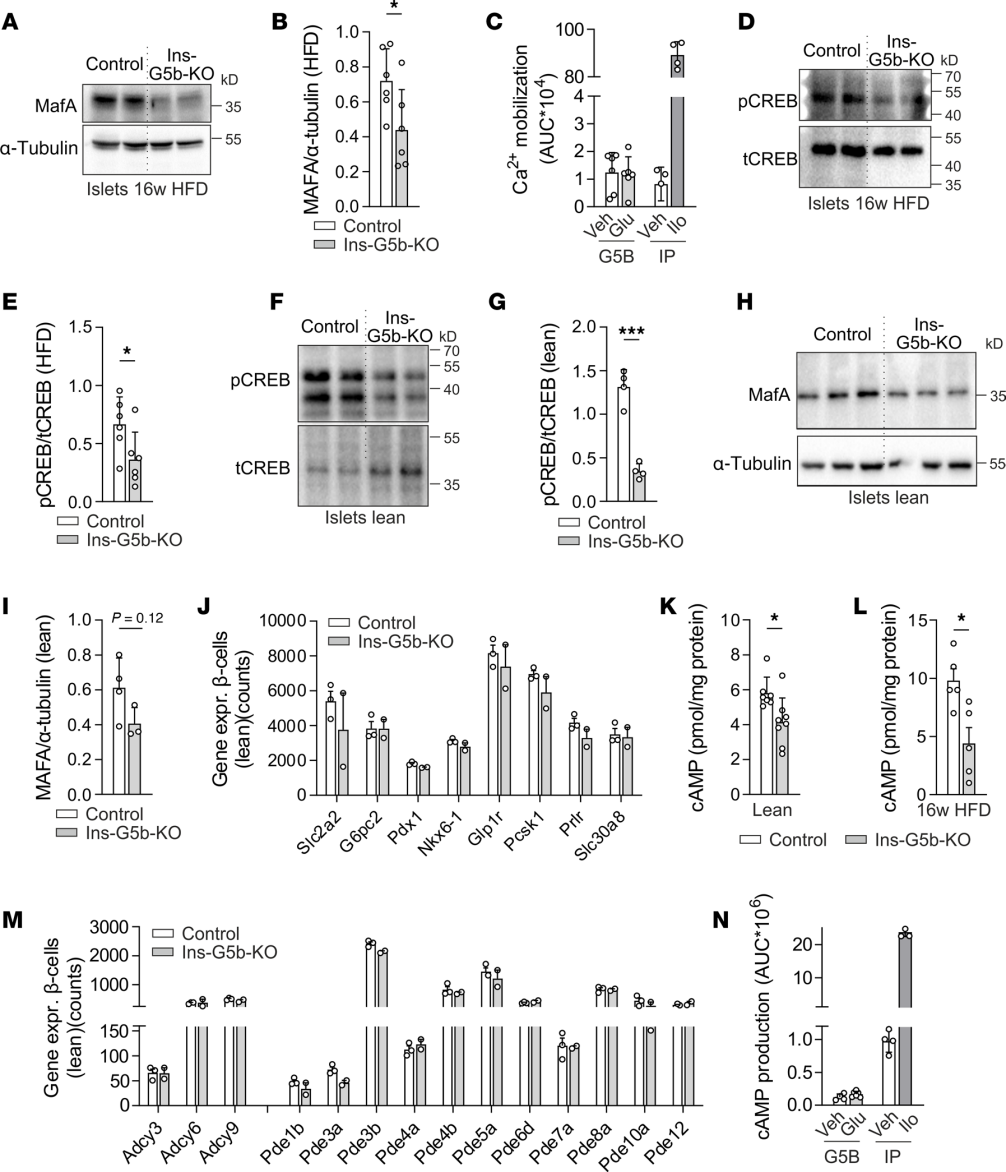
Impaired cAMP/CREB/MafA signaling in obese Ins-G5b–KOs. (**A** and **B**) Immunoblot analysis of MafA expression in lysates of islets harvested from obese control and Ins-G5b–KO mice: representative Western blot images (**A**) and statistical evaluation of band intensities (**B**, *n* = 6/6 mice). (**C**) Effect of 20 mM glucose (Glu) on calcium mobilization in HEK cells transfected with GPRC5B (G5B), a promiscuous G-protein, and a calcium sensor; iloprost-mediated (Ilo-mediated) stimulation of prostacyclin receptor IP-transfected cells serves as positive control (*n* = 6 G5B-Veh; *n* = 6 G5B-Glu; *n* = 4 IP-Veh; *n* = 4 IP-Glu). (**D**–**G**) Immunoblot analysis of basal CREB phosphorylation in islets isolated from HFD-fed (**D** and **E**, *n* = 6/6) or lean (**F** and **G**, *n* = 4/4) mice; total CREB as loading control. (**H** and **I**) Immunoblot analysis of MafA expression in islets harvested from lean mice; α-tubulin was used as loading control (*n* = 4/3 mice). (**J**) Expression of MafA target genes was determined by mRNA-Seq of flow cytometry–isolated β cells harvested from lean control and Ins-G5b–KO mice (*n* = 3/2 mice). The *y* axis represents DESeq2 normalized counts. (**K** and **L**) Basal cAMP levels in islets from lean (**K**) and obese (**L**) Ins-G5b–KOs following overnight culture in complete RPMI-1640 media (11.1 mM glucose) (*n* = 7/8 and 5/5 mice). (**M**) Expression of genes related to cAMP production/breakdown was determined by mRNA-Seq in flow cytometry–isolated β cells harvested from lean mice (*n* = 3/2 mice). The *y* axis represents DESeq2 normalized counts. (**N**) Effect of 20 mM glucose (Glu) on cAMP production in GPRC5B-overexpressing (G5B-overexpressing) HEK cells was determined using a GloSensor-based cAMP assay; iloprost-mediated (Ilo-mediated) stimulation of prostacyclin receptor IP-transfected cells serves as positive control (*n* = 4, 6, 4, 4). Data are mean ± SEM; comparisons between control and KO samples were performed using unpaired Student’s *t* test (**B**, **C**, **E**, **G**, **I**, **K**, **L**, and **N**) and multiple unpaired Student’s *t* test with 2-stage linear step-up procedure of Benjamini, Krieger, and Yekutieli (**J** and **M**). n, number of mice per group/individual wells of cells. *P ≤ 0.05, **P ≤ 0.01, ***P ≤ 0.001.

Because altered MafA-dependent β cell maturation could underlie the observed impaired β cell function in obese Ins-G5b–KOs, we examined whether MafA expression was also reduced on the protein level and found this to be the case (Figure 5, A and B). We then investigated how GPRC5B contributes to MafA expression in β cells. Since MafA expression can be regulated by glucose (25), and since glucose has been suggested as a putative ligand for BOSS, a putative *Drosophila* homolog of GPRC5B (26), we tested whether glucose was able to induce signaling effects in GPRC5B-overexpressing HEK cells, here using calcium mobilization after cotransfection with a promiscuous G protein and a GFP-aequorin calcium sensor as a readout (27). These assays did not show any glucose-induced calcium mobilization in GPRC5B-overexpressing HEK cells (Figure 5C).

Since MafA expression is also regulated by cAMP/CREB signaling (23), and because GPRC5B has been implicated in the regulation of the cAMP/CREB pathway (14), we next investigated whether altered cAMP/CREB signaling in GPRC5B-deficient β cells might contribute to reduced MafA expression. In line with this hypothesis, we found that CREB phosphorylation was significantly reduced in islets of HFD-fed KO mice (Figure 5, D and E). To test whether decreased CREB signaling preceded reduced MafA expression, we studied protein expression in islets of lean mice and found a similar reduction in CREB phosphorylation (Figure 5, F and G). This was associated with a trend to reduced MafA expression (Figure 5, H and I), but this mild change in MafA expression did not yet affect target gene expression in the lean state (Figure 5J). In line with reduced basal CREB phosphorylation, we found that basal cAMP levels were diminished in islets from lean and obese Ins-G5b–KOs (Figure 5, K and L). Acute cAMP production induced by high glucose and GLP-1, an agonist at the G_s_-coupled GLP-1 receptor, however, was not altered (Supplemental Figure 5A). As to the mechanism underlying reduced basal cAMP levels in the absence of GPRC5B, we did not observe differences in enzymes involved in cAMP production or breakdown (Figure 5M), and studies in GPRC5B-overexpressing HEK cells did not reveal a direct, GPRC5B-dependent effect of glucose on cAMP production (Figure 5N). Also in β cells from obese Ins-G5b–KOs, single-cell RNA-Seq did not reveal relevant changes in the expression of heterotrimeric G-proteins, adenylyl cyclases and phosphodiesterases, or GPCRs related to insulin secretion (Supplemental Figure 5, B–D). These findings collectively suggest that loss of GPRC5B in β cells leads through a yet unknown mechanism to decreased cAMP production, which in turn results in diminished phosphorylation of CREB and reduced expression of MafA. Consequently, this cascade of events impairs the MafA-dependent maintenance of the mature β cell phenotype.

## Discussion

Previous expression studies suggested that orphan GPCR GPRC5B is upregulated in pancreatic islets of patients with type 2 diabetes (16), but the in vivo function of GPRC5B in β cells has not been thoroughly addressed. Here, using a β cell–specific conditional KO mouse model, we show that GPRC5B regulates β cell function through cAMP/CREB-dependent regulation of MafA expression.

So far, the function of GPRC5B in metabolic processes has mainly been studied in the context of adipose tissue biology (28); global GPRC5B-KO mice were protected from diet-induced obesity and insulin resistance, and metabolic-induced inflammation in their white adipose tissue was reduced (13). Circulating insulin levels were decreased in global GPRC5B-KOs (13, 17), but these changes were mainly attributed to their improved insulin sensitivity. Whether or not GPRC5B directly contributes to β cell function in vivo, in particular to insulin secretion, was so far not clear. Lentiviral shRNA-mediated knockdown of GPRC5B in cultured mouse islets enhanced basal and glucose-induced insulin secretion, but which islet cell types were responsible for these changes was not evaluated (16). The same study showed reduced cytokine-induced cell death in cells isolated from *Gprc5b*-knockdown islets (16), whereas overexpression of GPRC5B in MIN6 insulinoma cells enhanced both proliferation and apoptosis (18). Our data show that loss of GPRC5B expression in β cells did not have clear effects on viability or the expression of proliferation or apoptosis markers in the lean state; genes associated with insulin production or secretion were not altered. In line with this, basal and glucose-induced insulin secretion was normal in lean mice. The differences between the above-mentioned in vitro studies and our results are most likely due to the different cell types analyzed. MIN6 cells, for example, differ from primary β cells in key aspects such as GSIS, proliferation, gene expression, and cellular heterogeneity, making them less physiologically representative (29, 30). Additionally, their tumor-derived nature may lead to genetic instability and altered signaling pathways, limiting their translational relevance.

While β cell function was not affected in the lean state, induction of obesity by HFD resulted in clearly reduced insulin secretion in Ins-G5b–KOs. As an underlying mechanism, we identified a reduction of cAMP/CREB-dependent MafA expression in GPRC5B-deficient β cells, resulting in partial loss of the mature phenotype and, consecutively, reduced insulin secretion. MafA is a transcription factor crucial for the maturation and function of pancreatic β cells both during embryonic development and postnatal life; its expression decreases with age and chronic metabolic stress (22, 31). In adult mice, loss or dysfunction of MafA impairs the ability of β cells to maintain their mature phenotype, resulting in a reduced β cell/α cell ratio, impaired insulin production, and β cell failure in diabetes (22, 31). MafA regulates the expression of insulin and other genes essential for GSIS — for example, glucose transporter GLUT-2 (encoded by *Slc2a2*) or cation channel TRPM5 (22). MafA expression is regulated by a number of transcription factors, such as Foxa2, Nkx2.2, Pdx1, Nkx6.1, Pax6, and Hnf1a (22, 23), but we did not observe significant changes in the expression of these factors. Another regulator of MafA expression is the transcription factor CREB, a crucial effector of the G_s_/cAMP/PKA signaling cascade, and CREB binding sites were identified both in MafA promoter and enhancer regions (32, 33). Herein, we demonstrate that GPRC5B deficiency in β cell results in decreased cAMP production and impaired CREB phosphorylation, and we hypothesize that these changes contribute to reduced MafA expression. Interestingly, MafA levels are — despite reduced CREB phosphorylation — not yet significantly changed in lean mice, which might indicate that GPRC5B-dependent cAMP/CREB regulation becomes more relevant for MafA regulation during obesity development. Alternatively, it is possible that GPRC5B contributes to MafA regulation not only through cAMP/CREB but also through yet-unknown CREB-independent pathways, which are particularly relevant in the obese state.

In addition to modulating MafA expression, cAMP/CREB signaling has been implicated in the regulation of β cell proliferation and survival — for example, by regulating expression of insulin receptor substrate 2 or regulators of apoptosis such as Bcl-2 and Bax (34, 35). However, we did not observe altered viability in cultured islets from Ins-G5b–KOs, suggesting that the reduction of cAMP/CREB signaling was either not strong enough to disturb CREB-dependent survival pathways or that other pathways were able to compensate these changes. In addition to its role in β cell survival, cAMP plays an important role in insulin secretion; cAMP elevation triggered by activation of G_s_-coupled receptors such as the GLP-1 receptor facilitates glucose-induced insulin secretion in a PKA/Epac2-dependent manner (4). Interestingly, though basal cAMP levels were reduced in cultured islets from Ins-G5b–KOs, acute cAMP production induced by glucose and GLP-1 was normal.

The mechanisms by which GPRC5B modulates cAMP production in resting islets are not understood, and it is unclear whether a GPRC5B ligand is involved. Currently, no endogenous GPRC5B ligands are known, but *Drosophila* receptor BOSS, which shows some degree of homology with GPRC5B, was shown to respond under certain conditions to glucose, at least in receptor-overexpressing HEK cells (26). We tested whether similar findings could be observed for GPRC5B but failed to detect glucose responses in transfected HEK cells both in calcium and cAMP assays. As to other mechanisms of activation, some orphan GPCRs are known to modulate cAMP production in a constitutive, ligand-independent manner, but neither own data nor published studies (36) show constitutive activity of overexpressed GPRC5B toward G_s_ family G-proteins or other G-protein families. In other cell types, for example in smooth muscle cells or macrophages, GPRC5B has been shown to modulate cAMP production through dimerization with prostanoid receptors such as the prostacyclin receptor IP or the PGE_2_ receptor EP2 (14, 15), and it is possible that this mechanism also plays a role in β cells. β Cells express a large number of receptors that are able to modulate cAMP levels — for example, the G_s_-coupled receptors for GLP-1, glucose-dependent insulinotropic polypeptide, or pituitary adenylate cyclase-activating polypeptide. Also G_i/o_-coupled receptors such as the α_2_-adrenergic receptor or acetate receptors FFA2 and FFA3 modulate cAMP levels, and together, they regulate insulin secretion and β cell survival/maturation through cAMP-dependent activation of protein kinase A and Epac2 (4). Furthermore, the prostanoid receptor IP (but not EP2) is expressed in β cells (37), but whether GPRC5B dimerization with IP exists in these cells and contributes to modulation of cAMP levels is currently unknown.

Taken together, our data show that GPRC5B helps to maintain a mature β cell phenotype by regulating the cAMP/CREB/MafA pathway and that genetic inactivation of this receptor leads to reduced insulin secretion and glucose intolerance in HFD-fed mice. In this context, it is interesting to note that GPRC5B expression is increased in islets of patients with type 2 diabetes (16). We therefore propose that upregulation of GPRC5B under conditions of metabolic stress represents a potentially new regulatory mechanism for maintaining mature β cell function and, consecutively, glucose homeostasis.

## Methods

### Sex as a biological variable

In our in vitro studies we used islets from both male and female mice. Our in vivo studies examined male mice because male animals exhibited less variability in phenotype.

### Experimental animals

For the generation of tamoxifen-inducible, β cell–specific GPRC5B knockouts (Ins-G5b–KOs), mice carrying a floxed *Gprc5b* allele (15) were intercrossed with the *Ins1*-CreERT2 mouse line (Tg[Ins1-cre/ERT2]16.7Ics) (19). *Ins1*-CreERT2 mice contain a Cre recombinase/tamoxifen-inducible estrogen receptor fusion (Cre/ERT2) gene driven by the *Ins1* (insulin I) promoter and were obtained as breeding stocks from Institut Clinique de la Souris, France. Immunofluorescence staining in *Ins1*-CreERT2 mice crossed to the Cre reporter line mTmG (20) revealed that 99.2±0.27% of insulin-expressing cells were Cre positive, whereas only 1.29±0.61% of Cre-positive cells were insulin-negative.

Breeding of experimental animals was performed by intercrossing hemizygous *Ins1*-CreERT2-positive *Gprc5b*^fl/fl^ mice with Cre-negative *Gprc5b*^fl/fl^ mice, resulting in equal numbers of Ins-G5b–KOs and Cre-negative *Gprc5b*^fl/fl^ littermate controls. Mice were kept on a C57BL6 background that was predominantly C57BL/6J, with a minor contribution of C57BL/6N.

To facilitate the flow cytometric isolation of β cells, some mice were also harboured a Cre-dependent fluorescent reporter construct mTmG (Rosa26flox-mT-stop-flox-mG) (20). Genotyping for Gprc5b was performed using the primers 5′-GCTGGAAGGTTTCTCCCTCT-3′ and 5′-AAGAGACAACCACCAGACAGG-3′, which resulted in band sizes of 361 for the wild-type allele and 478 bp for the floxed allele.

For induction of Cre-mediated recombination, mice were treated with 1 mg tamoxifen (Sigma, T5648) i.p. for 5 consecutive days. All experiments were performed in male mice at an age of 8-28 weeks. If not otherwise indicated, experiments were performed 2-3 weeks after the end of tamoxifen induction. Mice were housed under a 12:12-hour light-dark cycle with free access to water and food and under pathogen free conditions.

### Animal models

For diet-induced obesity experiments, mice were fed for 16 weeks with a Western-style HFD (30% crude fat, 21% crude protein, 16.9% sugar, 16.3% starch, 5.4% crude ash and 5% crude fiber; Ssniff, E15126-34).

#### GTT.

Mice were fasted (water ad libitum) for 6-12 hours (6 hours for lean mice, 12 hours for obese mice). For the GTT, mice received 1.5-2 g/kg glucose (2 g/kg for lean mice, 1.5 g/kg for obese mice) i.p. for i.p. GTT or by gavage for oral GTT. Blood was collected by puncture of a distal tail vein, and the blood glucose concentrations were measured using a glucose meter (Accu-Check Sensor, Roche) before and 15, 30, 60, 90, and 120 minutes after dosing.

#### GSIS assay.

As described above for GTT, mice were fasted for 6-12 hours and glucose was administered. Blood was then obtained from the tail tip before and 5, 15, and 30 minutes after glucose administration, collected in EDTA-coated tubes (Sarstedt, Microvette CB 300 K2E), centrifuged at 2,000*g* for 15 minutes to obtain plasma. Plasma insulin levels were determined using mouse insulin ELISA kit (Mercodia, 10-1247-01) according to the manufacturer’s instructions.

#### ITT.

Mice were fasted for 4-6 hours. For the ITT, mice were administered 0.75 unit/kg insulin (SANOFI, Insuman Rapid 100IE) i.p. Blood was collected by puncture of a distal tail vein, and blood glucose concentrations were measured using a glucose meter (Accu-Check Sensor, Roche) before and 15, 30, 60 and 90 minutes after dosing.

#### Plasma gastrointestinal hormone level determination.

To determine plasma levels of metabolic-related hormones in HFD-fed mice, blood was collected from the tail tip of fed or 12-hour fasted mice using EDTA-coated tubes (Sarstedt, Microvette CB 300 K2E). The tubes were then placed in a centrifuge at 2,000*g* for 15 minutes to obtain plasma. Hormone levels were subsequently measured using the MILLIPLEX Mouse Metabolic Hormone Multiplex Assay (Merk, MMHE-44K) and the Luminex xMAP MAGPIX platform, following the manufacturer’s instructions.

### Isolation and culture of murine islets

Following the euthanasia of tamoxifen-treated mice, the pancreas underwent perfusion and digestion with digestion buffer (HBSS buffer [Thermo Fisher Scientific, 14175129] supplied with 0.5% BSA, 20 mM HEPES and 1 mg/mL collagenase XI [Sigma, C7657]), at 37°C in a water bath for 11 minutes. To halt the enzymatic reaction, HBSS buffer (Thermo Fisher Scientific, 14175129) containing 0.5% BSA, 1 mM CaCl_2_, and 20 mM HEPES was added. Subsequently, the islets underwent 2 washes via centrifugation at 290*g*, 4°C for 30 seconds, and were then filtered through a 70 μm cell strainer, with the filtrate being discarded. The islets retained on the cell strainer were carefully transferred to a petri dish and handpicked under a microscope. The isolated islets were cultured overnight in RPMI-1640 media (Thermo Fisher Scientific, 21875091) supplemented with 10% FBS and 1% penicillin and streptomycin. Cultures were maintained at 37°C in a humidified atmosphere with 5% CO_2_ before conducting a functional assay.

### GSIS in isolated murine pancreatic islets

On the day after isolation, islets were picked and preequilibrated in a 2.8 mM glucose HBSS buffer in a 6-well suspension culture plate (Greiner Bio-One, 657185) for 30 minutes at 37°C. Thereafter, 5 islets were transferred into a 1.5 mL reaction vessel containing 500 μL of 2.8 mM glucose HBSS buffer and incubated at 37°C for 15 minutes with 200 rpm shaking. After removal of the supernatant, 500 μL of 37°C-equilibrated 2.8 mM glucose HBSS, 16.7 mM glucose HBSS, and 30 mM KCl were added in sequence. For each addition, samples were incubate at 37°C while shaking at 200 rpm for 30 minutes, then supernatant was collected for insulin level determination. Afterward, the islets were collected and lysed in an acid ethanol solution (96% ethanol supplied with 0.18M HCl) for total insulin content extraction. The insulin level was determined by mouse insulin ELISA kit (Mercodia, 10-1247-01) following the manufacturer’s instructions. Insulin values were quantified and normalized to total insulin content.

### Calcium mobilization in isolated murine pancreatic islets

On the day after isolation, islets were washed twice with 2.8 mM glucose HBSS buffer, before being transferred into 2.8 mM glucose HBSS containing 10 μM of Fluo-8 AM (AAT Bioquest, 21081) and incubated for 30 minutes at 37°C. Subsequently, the islets were washed twice with 2.8 mM glucose HBSS buffer and then incubated in a poly-L-Lysine (0.1 mg/mL, Sigma, P9155) coated chambered coverslip (Ibidi, 81816) for 15 minutes at 37°C before imaging to allow for efficient Fluo-8 AM cleavage. Islet imaging was performed using a fluorescence microscope (Zeiss, Observer Z1) equipped with a green fluorescent protein (GFP) filter set. The fluorescence signal was recorded at 5-second intervals for a total of 20 minutes. At the 3-minute mark, 45 mM glucose solution was added to the chamber to achieve a final glucose concentration of 16.7 mM. The resulting data are expressed as the mean fluorescence intensity within the islet area, which was quantified using Fiji ImageJ (NIH).

### cAMP assays in isolated murine pancreatic islets

cAMP levels were measured using the Direct cAMP ELISA kit (Enzo, ADI-900-066A) according to the manufacturer’s protocol. In brief, murine islets were isolated and cultured overnight in RPMI-1640 media (11.1 mM glucose) supplied with 10% FBS and 1% penicillin and streptomycin (complete RPMI-1640 media), at 37°C in a humidified atmosphere with 5% CO_2_. For basal cAMP determination, 20–25 healthy islets were selected and equilibrated with complete RPMI-1640 media containing 50 μM phosphodiesterase inhibitor IBMX (Tocris Bioscience, 2845) for 60 minutes. Thereafter, the islets were transferred into the same media and incubated at 37°C for another 30 minutes before sample harvesting. For acute agonist-induced cAMP production, 20–25 healthy islets were starved in 2.8 mM glucose complete RPMI-1640 media (prepared from Thermo Fisher Scientific, 11879020) for 30 minutes, followed by a 30-minute equilibration in the same media supplemented with 50 μM IBMX. They were then stimulated with 16.7 mM glucose in complete RPMI-1640 media, along with 100 nM GLP-1 and 50 μM IBMX, for another 30 minutes until sample harvesting. By end of the 30-minute incubation, the solution was carefully discarded, and the islets were lysed and mechanically homogenized in 130 μL HCl (0.1 M) containing 0.1% Triton X-100. The islets were subsequently subjected to centrifugation at 1,000*g* for 10 minutes to remove cellular debris. The collected supernatants were then assessed for cAMP levels using the acetylation format, following the manufacturer’s instructions. Protein concentration was determined using a BCA Protein Assay (Thermo Fisher Scientific, 23227), and data were normalized to protein amount (cAMP pmol/mg of protein).

### LDH cytotoxicity assay

Murine islets were cultured in complete RPMI-1640 in the presence or absence of a cytokine cocktail (100 ng/mL IL-1β, 125 ng/mL TNF-α, and 125 ng/mL INF-γ) for 24 hours at 37°C in a humidified atmosphere with 5% CO_2_. Supernatants were then collected and assayed for LDH release using the LDH cytotoxicity assay kit (Invitrogen, C20300). Absorbance was measured at 490 nm and 680 nm. To eliminate background signals from the instrument, the absorbance at 680 nm was subtracted from the absorbance at 490 nm to obtain absolute values. The data presented are these absolute values, with the subtraction of values from the culture media alone (without islet cells) groups.

### Transmission electron microscopy (TEM)

For TEM imaging of pancreatic islet sections, freshly isolated islets were initially mixed 1:1 with prewarmed, double-concentrated fixative in culture medium, consisting of 4% paraformaldehyde (PFA) and 5% glutaraldehyde (GA) in 0.2 M cacodylate buffer (pH 7.4). The samples were incubated for 5 minutes at 37°C. Subsequently, the islets were transferred to fresh fixative (2% PFA + 2.5% GA in 0.1M cacodylate buffer, pH 7.4) and incubated at room temperature for 2 hours. After fixation, the islets were embedded in 3% low melting point (LMP) agarose. Small agarose gel blocks were prepared for further processing. Samples then were rinsed with 0.1 M cacodylate buffer (pH 7.4), postfixed with 1% (w/v) osmium tetroxide (OsO_4_) in distilled water for 1 hour and washed with distilled water. En bloc staining was performed using 2% aqueous uranyl acetate for 1 hour. The specimens were dehydrated through a graded ethanol series and subsequently transferred to propylene oxide. Embedding was carried out in Epon resin according to established protocols. Ultrathin sections (60 nm) were cut using a Leica UC7 ultramicrotome and mounted on 2 × 1 mm copper slot grids. The sections were postcontrasted with uranyl acetate and lead citrate. Sections were examined using a JEM-1400 Plus transmission electron microscope (Jeol, Japan), operated at an accelerating voltage of 120 kV. Digital images were acquired using an EM-14800Ruby CCD camera system (3,296 × 2,472 pixels). The area and diameter of mitochondria were determined using ImageJ software (NIH).

### EdU staining in islets

For in vivo EdU incorporation, mice received s.c. injections of 50 μL of a 20 mg/mL EdU solution (Lumiprobe, 40540) every other day from days 9 to 1 before organ harvest. Islets were isolated and fixed in 4% PFA for 10 minutes at 4°C, OCT-embedded, and cryosectioned at 5 μm onto Superfrost plus slides (Epredia, J1800AMNZ). EdU signals were revealed using the Click-iT EdU Imaging Kits (ThermoFisher, C10340) in combination with DAPI staining following the manufacture’s instruction. Slides were then incubated with anti-insulin-AF488 antibody (Cell Signaling Technology, 9016S) at 1:1,000 dilution overnight at 4°C. Following three 5-minute PBS washes, the slides were mounted. Images were acquired using a Leica Thunder Imager and analyzed with ImageJ.

### Caspase-3/7 activity in islets

For flow cytometric measurement of Caspase-3/7 activity, isolated islets were dissociated into single cells by incubating with Accutase at 37°C for 30 minutes. The enzyme reaction was terminated by adding an equal volume of culture media. After centrifugation at 300*g* for 4 minutes, islet cells were resuspended and incubated with culture media, with or without 10 μM H_2_O_2_, for 6 hours at 37°C. Cells were then incubated with 5 μM BioTracker NucView 405 Blue Caspase-3 Dye (Sigma, SCT104) for 15 minutes at room temperature, followed by 2 PBS washes at 300*g* for 4 minutes. Cells were then incubated with 7-AAD (SYTOX AADvanced Dead Cell Stain Kit, ThermoFisher, S10349) at 1:1,000 for 15 minutes. After two 4-minute PBS washes at 300*g*, cells were fixed in fixation buffer (4% PFA, 0.1% saponin in PBS) for 15 minutes at 4°C and incubated with anti–insulin-AF488 antibody (Cell signaling technology, 9016S) at 1:100 dilution for 30 minutes at room temperature. Afterward, cells were washed with PBS by spinning down at 300*g* for 4 minutes, resuspended, and filtered through a 40 μm filter into FACS tubes (38). After doublet discrimination, the contribution of the 7-AAD–negative, NucView 405–positive cell population to the total percentage of insulin-positive cells was determined using a BD DACS Canto II.

### ROS level determination

For flow cytometric evaluation of ROS levels, isolated pancreatic islets were dissociated into single cells by incubating with Accutase (Innovative Cell Technologies, AT-104) at 37°C for 30 minutes. The enzyme reaction was terminated by adding an equal volume of culture media. After centrifugation at 300*g* for 4 minutes, islet cells were resuspended and incubated with culture media, with or without 200 μM H_2_O_2_, for 30 minutes at 37°C. This was followed by incubation with Cellular ROS Assay Kit (Deep Red, Abcam, ab186029) for 1 hour at 37°C. After spinning down at 300*g* for 4 minutes to remove excess ROS indicator, cells were incubated with DAPI (4’,6-Diamidino-2-Phenylindole, Thermofisher, D3571) for 5 minutes, washed again, and fixed in fixation buffer (4% PFA, 0.1% saponin in PBS) for 15 minutes at 4°C. Cells were then incubated with anti–insulin-AF488 antibody (Cell signaling technology, 9016S) at 1:100 dilution for 30 minutes at room temperature. Subsequently, cells were washed with PBS by spinning down at 300*g* for 4 minutes, resuspended, and filtered through a 40 μm filter into FACS tubes (38). Samples were analyzed using a BD Canto II flow cytometer. After doublet discrimination, the DAPI-negative population was gated, and the mean fluorescence intensity (MFI) of ROS indicator (deep red) were determined in insulin-positive populations.

### Aequorin-based calcium mobilization in HEK293 cells

Aequorin-based calcium mobilization assays were performed as previously described (27). Briefly, HEK293 cells were transfected in a poly-l-lysine–coated, clear-bottomed, white-walled 96-well plate (PerkinElmer, Viewplate-96TC) with expression plasmids encoding a calcium-sensitive bioluminescent fusion protein (27), a promiscuous G protein, and indicated GPCRs using Lipofectamine 2000. Two days after transfection, cells were washed twice and then loaded with 50 μL of coelenterazine-h solution (5 μM, Promega, S2011) in Hank’s balanced salt solution (HBSS, contains no glucose for GPRC5B/glucose group) with calcium and magnesium containing 10 mM HEPES for 2 hours at 37°C, 5% CO_2_. For ligand dispensing, 2X ligand solutions (40 mM glucose or 2 μM iloprost) were prepared with HHBS++ buffer (calcium and magnesium supplied HBSS buffer which contains 10 mM HEPES) in V-bottom 96-well plates and equilibrated to room temperature prior to reading. The auto-dispensing function of the Flexstation3 was used to dispense 50 μL volumes of each ligand-containing solution into the cell plates. The luminescence signal was recorded and analyzed using SoftMax Pro 7 software (Molecular Devices).

### F22-GloSensor-based cAMP measurement

To determine cAMP levels in cells, HEK293 cells stably carrying a F22 cAMP GloSensor (Promega, E2301) were transfected with the appropriate receptor construct using Lipofactamine 2000. Two days after transfection, the cell culture was replaced with glucose-free DMEM media (Thermo Fisher Scientific, 11966025) containing GloSensor cAMP reagent (Promega, E1290) and incubated for another 2 hours. The appropriate ligand solutions were then added to the cells to achieve the desired concentration. After 15 minutes, luminescence was measured using Flextation 3 (Molecular Devices).

### Histological and immunohistochemical analyses of murine tissues

For histologic analysis of murine pancreas, pancreas was fixed in 4% PFA (in PBS) overnight, dehydrated and embedded in paraffin. Serial sections (5 μm) were cut transversely through the tissues using a rotary microtome (Thermo Scientific, Microm HM355S) and mounted on Superfrost plus slides (Epredia). After deparaffinization and rehydration, the collected sections were stained using a standard hematoxylin and eosin (H&E) staining protocol, with hematoxylin solution (Roth, T865.2) and eosin solution (Roth 3137.1).

For insulin immunostaining of pancreatic sections, the collected sections were deparaffinized and rehydrated, then endogenous peroxidases were quenched with 3% H_2_O_2_ solution in water for 30 minutes. After 20 minutes of incubation in 2.5% horse serum in PBS, the sections were incubated with rabbit anti-mouse insulin antibody (Cell Signaling Technology, 3014S) diluted 1:1,000 in 2.5% horse serum in PBS overnight at 4°C. The next day, after three 5-minute washes with PBS, the sections were incubated with biotinylated secondary antibody (ABC Elite Kit, Vector, PK-166100) for 30 minutes, followed by three 5-minute washes. The staining signal was amplified using the ABC Elite Kit, followed by peroxidase detection by incubation with DAB substrate (3,3’-diaminobenzidine, Vector, SK-4100), after which the sections were counterstained with hematoxylin and dehydrate and mounted. Images were captured with a microscope (Zeiss) and analyzed with Fiji ImageJ (NIH).

### Total insulin content in pancreas

The pancreas was carefully dissected, snap-frozen in liquid nitrogen, and weighed. To extract the total insulin content, the pancreas tissues were thoroughly homogenized in a chilled acid ethanol solution (96% ethanol supplied with 0.18M HCl), and samples were placed on dry ice between each homogenization interval to avert overheating. Following centrifugation at 17,000*g* for 30 minutes at 4°C, the supernatant was collected and diluted for insulin level determination using a mouse insulin ELISA kit (Mercodia, 10-1247-01) according to the manufacturer’s instructions.

### Western blotting of tissue samples

Samples were lysed in RIPA lysis buffer (Thermo Fisher Scientific, 89900) (25 mM Tris-HCl pH 7.6, 150 mM NaCl, 1% NP-40, 1% sodium deoxycholate, 0.1% SDS) supplemented with protease and phosphatase inhibitors (Thermo Fisher Scientific, 78440). Proteins were separated by SDS-PAGE (Tris-glycine gels with Tris/glycine/SDS buffer) and transferred onto nitrocellulose membranes (Amersham, 10600003) using the Mini Trans-Blot Cell (Bio-Rad). The membrane were then blocked with 5% bovine serum albumin (BSA) in Tris-buffered saline with 0.1% Tween 20 detergent (TBST) for 40 minutes at room temperature. Thereafter, blots were probed at 4°C overnight with specific primary antibodies as indicated. After six 5-minute washes in TBST, the membranes were incubated with peroxidase-conjugated secondary antibodies (dilution 1:3000 in 5% BSA/TBST) for 1 hour at room temperature. The following primary antibodies were utilized: rabbit anti phospho-CREB (Ser133) (Cell Signaling Technology, 9198s, 1:750), rabbit anti total CREB (Cell Signaling Technology, 9197, 1:1000), rabbit anti MAFA (Thermo Fisher Scientific, A300-611A, 1:1000), rabbit anti GAPDH (Cell Signaling Technology, 2118, 1:1000), rabbit anti α-Tubulin (Cell Signaling Technology, 2125, 1:1000). Secondary antibodies used were horseradish peroxidase-conjugated anti-rabbit IgG (Cell Signaling Technology, 7074V). Antibody binding was revealed using enhanced chemiluminescence reagent (Thermo Fisher Scientific, 32106 for normal sensitivity; Millipore, WBKLS0500 for ultra-sensitivity) and ChemiDoc MP Imaging System (Bio-Rad). Band intensities were quantified using the mean gray value method in Fiji ImageJ software (NIH).

### Expression analysis

RNA isolation from cell lines was performed using the Quick-RNA Microprep Kit (Zymo, R1051), and RNA extraction from tissues and flow cytometry–sorted primary cells was done using the RNeasy micro kit (Qiagen, 74004), both combined with on-column DNase digestion to avoid contamination by genomic DNA. Reverse transcriptions were carried out using ProtoScript II First Strand cDNA Synthesis Kit (New England Biolabs, E6560) following the manufacturer’s standard protocol. Primers for qPCR were as follows:

Mu *Gapdh*: forward (5′ to 3′), TGGCCTTCCGTGTTCCTAC; reverse, GAGTTGCTGTTGAAGTCGCA; Mu *Gprc5b*: forward, CGCTGCAGAGATGTGACTTG, reverse, TCTCTAACACCAGGAACATTCG.

Transcript quantification was performed using the Power SYBR Green PCR Master Mix (Thermo Fisher Scientific, 4367659) and the QuantStudio 1 Real-Time PCR System (Thermo Fisher Scientific). Gene expression was normalized to the endogenous control (*Gapdh*) and calculated using the ΔΔCt method.

For mRNA-Seq, EGFP-expressing pancreatic β cells were isolated from tamoxifen-induced control and Ins-G5b–KOs carrying the mTmG reporter strain (20). To do so, isolated mouse islets were digested with Accutase solution (Innovative Cell Technologies, AT-104) at 37°C for 30 minutes with 300 rpm shaking. The digestion procedure was halted by the addition of complete culture media, and then the cell suspension was filtered through a 40 μm cell strainer. Following centrifugation at 600*g* for 5 minutes at 4°C, the supernatant were carefully aspirated. This washing step was repeated twice. After the last wash, cell pellets were carefully suspended in cold HBSS containing 1% BSA and DAPI for dead cell exclusion in flow cytometry. EGFP-positive cells were subsequently sorted into 1.5 mL reaction tubes containing 0.5 mL cold DMEM/PBS (v/v 1:1) using BD FACSMelody. The collected cells were then spun down at 600*g* for 5 minutes at 4°C. The resultant supernatant was removed, and RNA was isolated as described above. RNA and library preparation integrity were verified with LabChip Gx Touch (Perkin Elmer). RNA amounts were normalized and 1 μg of total RNA was used as input for VaZyme_VAHTS Universal Stranded mRNA-seq - V6 (Vazyme). Sequencing was performed on the NextSeq2000 platform (Illumina) using P3 flowcell with 72 bp single-end setup. The mRNA-Seq data are presented as DESeq2 normalized counts, calculated using DESeq2 median of ratios method.

The islet single-cell sequencing procedure was executed in accordance with the previously documented methodology (39). Briefly, the islets were isolated and dissociated using Accutase solution at 37°C for a duration of 30 minutes. Thereafter, the reaction was halted by the addition of complete culture media, and the solution was filtered through a 40 μm cell strainer. Centrifugation at 200*g* for 3 minutes 30 seconds at room temperature was then performed, with the resultant supernatant being discard. For antibody-based cell hashing, cell pellets were carefully suspended in staining buffer (PBS containing 2% BSA and 0.02% Tween). Subsequently, 1 μg of the designated rat anti-mouse hashing antibodies (BioLegend, TotalSeq anti-mouse Hashtag reagents) were incorporated and incubated for a duration of 30 minutes at 4°C. And then 2 mL of neutralizing solution (complete culture media/PBS, v/v 2:1) was added to the cell suspension, filtered through a 40 μm cell strainer, and sorted by BD FACSMelody for DAPI-based dead exclusion as well as doublet discrimination. The sorted cells were collected in a 1.5 mL reaction vessel containing 0.5 mL of neutralizing solution and spun down at 400*g* for 5 minutes at 4°C. The upper layer was then discarded, and an appropriate volume of solution was left to reach a final cell density of 500 cells per μL. The single cell sequencing and data analysis was performed as described previously (40). In brief, each sample-pool was run separately on a lane in Chromium controller with Chromium Next GEM Single Cell 3′ Reagent Kits v3.1 (10xGenomics). Single-cell RNA-Seq library preparation was done using standard protocol. Sequencing was performed on the Nextseq2000, with raw reads aligned against the mouse genome (mm10) and counted using StarSolo, followed by secondary analysis in Annotated Data Format and further analysis of preprocessed counts using Scanpy (40). Basic cell quality control was conducted by taking the number of detected genes and mitochondrial content into consideration. We removed 1,541 cells in total that did not express more than 300 genes or had a mitochondrial content greater than 8%. Furthermore, we filtered 14,472 genes if they were detected in less than 30 cells (<0.01%). Raw counts per cell were normalized to the median count over all cells and transformed into log space to stabilize variance. We initially reduced dimensionality of the dataset using PCA, retaining 50 principal components. Subsequent steps, including low-dimensional t-SNE embedding and cell clustering via community detection, were based on the initial PCA, with final data visualization performed using the CellxGene package as mentioned (40). KEGG pathway enrichment analysis was conducted using the KOBAS-i platform (41) focusing on differentially expressed genes (DEGs) with an absolute log_2_ fold change of 0.04 or greater.

### Cell culture and transfection

HEK293 cells (obtained from DSMZ, ACC-305) were cultured in Dulbecco’s modified Eagle’s medium (DMEM, Thermo Fisher Scientific, 10938025) supplemented with 10% fetal bovine serum (FBS, Thermo Fisher Scientific, 10270106), 1% penicillin and streptomycin (Thermo Fisher Scientific, 15140122), 1% L-glutamine (Thermo Fisher Scientific, 25030024), and 1% sodium pyruvate (Thermo Fisher Scientific, 11360070).

Transient transfections of HEK293 cells with expression vectors were carried out at 50%–80% confluency with Opti-MEM (Thermo Fisher Scientific, 31985062) and Lipofectamine 2000 transfection reagent (Invitrogen, 11668019) following the manufacturer’s instructions. The expression plasmid encoding GPRC5B (RC205201) was obtained from Origene, and the expression plasmid encoding PTGIR (PTGIR0TN00) was from cDNA.org.

### Statistics

All data are expressed as mean ± SEM. The following statistical tests were used: unpaired Student’s 2-tailed *t* test for comparisons between 2 groups, 2-way repeated measures ANOVA with Šidák’s multiple-comparison test for comparisons between 2 groups over time, and multiple unpaired Student’s *t* test with 2-stage linear step-up procedure of Benjamini, Krieger ,and Yekutieli for multiple comparisons between 2 groups. A *P* value less than 0.05 was considered significant.

### Study approval

All animal experiments were approved by the IACUC of the Regierungspräsidium Darmstadt and in accord with Directive 2010/63/EU of the European Parliament on the protection of animals used for scientific purposes.

### Data availability

The mRNA/single-cell RNA-Seq data have been deposited in the GEO database under accession no. GSE305589 and will become accessible upon publication. Values for all data points are reported in the Supporting Data Values file.

## Author contributions

TW performed most experiments, supported by JP (electron microscopy), SG (mRNA-Seq), and RB (calcium imaging, immunofluorescence staining). TW and NW designed the study, analyzed data, and wrote the manuscript.

## Supplementary Material

Supplemental data

Unedited blot and gel images

Supporting data values

## Version 1 09/04/2025

In-Press Preview

## Version 2 10/22/2025

Electronic publication
