# Supplementary material for: GPRC5B preserves a mature β cell state in obesity by controlling MafA expression
Source: JCI Insight. 2025 Sep 4;10(20):e194115. doi: 10.1172/jci.insight.194115 (PMC12581666; doi:10.1172/jci.insight.194115)
Supplement: Supplemental data [file jciinsight-10-194115-s152.pdf]

Supplementary Figures for Wang *et al*.

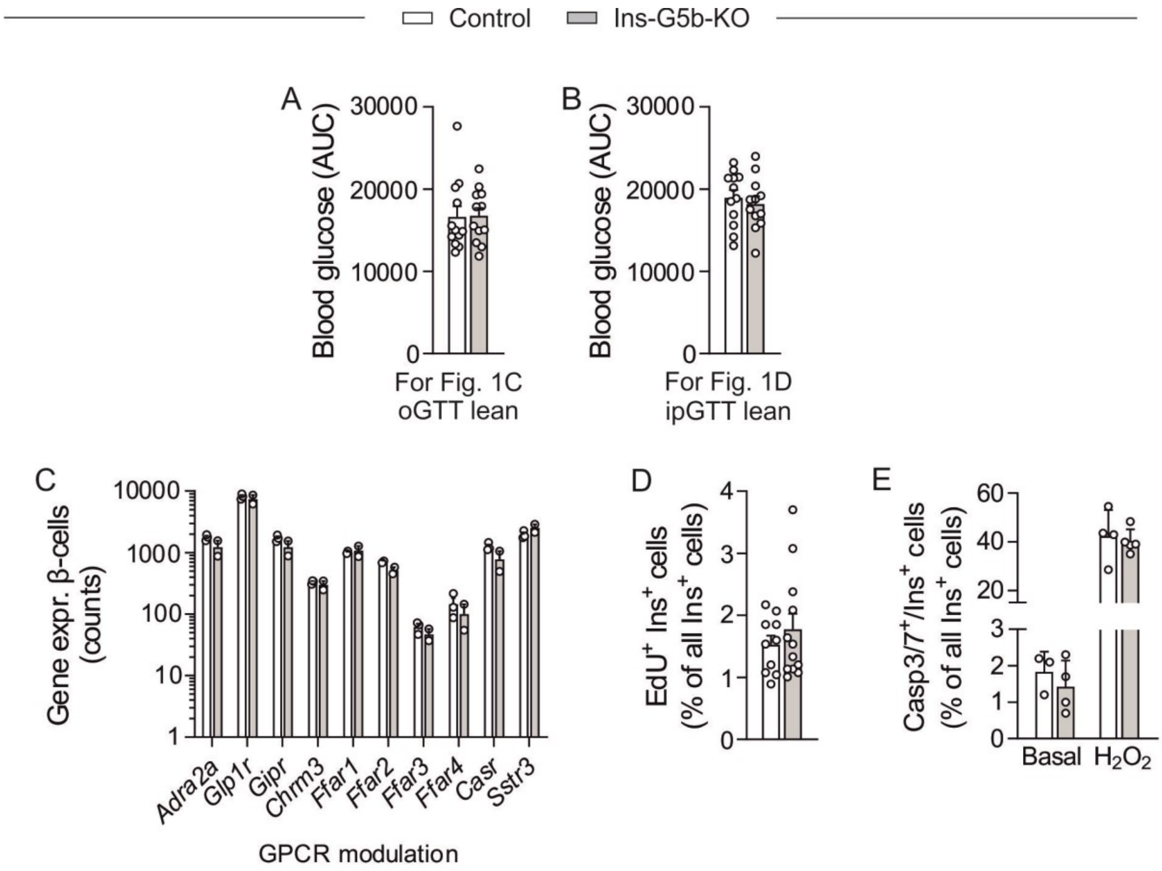

Supplementary Figure 1: **A,B,** Statistical evaluation of areas under the curve for glucose tolerance tests shown in Main text figures 1C (A) and 1D (B). **C,** Gene expression levels of G-protein-coupled receptors (GPCRs) with established roles in regulating β-cell function were assessed through mRNA sequencing (same samples as in main Fig. 1F) (n=3/2 mice). Y-axis represents DESeq2 normalized counts. **D,** The percentage of EdU-positive β-cells (EdU^+ Ins^+) was determined in pancreatic islets from EdU-pretreated control and Ins-G5b-KOs (n=10/12 islets from 3/3 mice). **E,** The percentage of Caspase-3/7 positive β-cells ($Casp3/7^+$; Ins^+) was determined in dissociated islet cells from control and Ins-G5b-KOs under basal conditions and after induction of apoptosis by 10 µM H_2O_2 for 6 hours (n=4/4 mice).

Data are means ± SEM; comparisons between groups were performed using unpaired, two-sided t test (A,B,D) or multiple unpaired Student's t-test with two-stage linear step-up procedure of Benjamini, Krieger and Yekutieli (C,E).

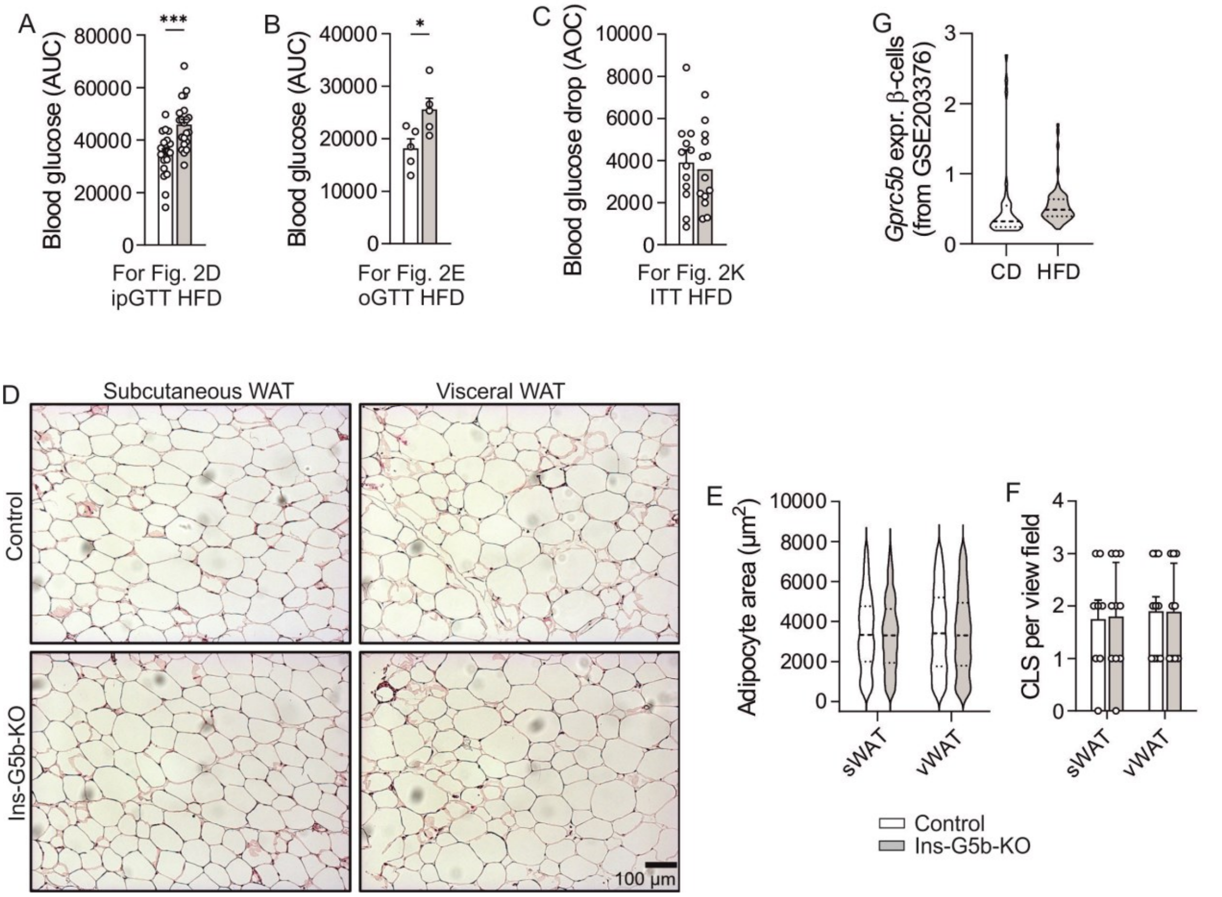

Supplementary Figure 2: A-C, Statistical evaluation of areas under the curve (AUC) for glucose tolerance tests shown in main figures 2D (A) and 2E (B) as well as of areas over the curve (AOC) for insulin tolerance test in main figure 2K (C). **D-F,** H&E staining of subcutaneous inguinal WAT (sWAT) and visceral epididymal WAT (vWAT) isolated from 16-week HFD-fed control and Ins-G5b-KO mice: Exemplary photomicrographs (D) and statistical evaluation of adipocyte area (E) or number of crown-like structures (CLS) (F). n=2/3 mice. Data are mean ± SEM. **G,** *Gprc5b* expression in β-cells of chow-fed (CD) mice and mice fed for 8 weeks with HFD (reanalysed from GSE203376).

Data are means ± SEM; comparisons between groups were performed using unpaired two-sided t test (A,B,C,G) or two-way ANOVA with Sidak's multiple comparisons test (E,F). *$P \leq 0.05$, ***$P \leq 0.001$.

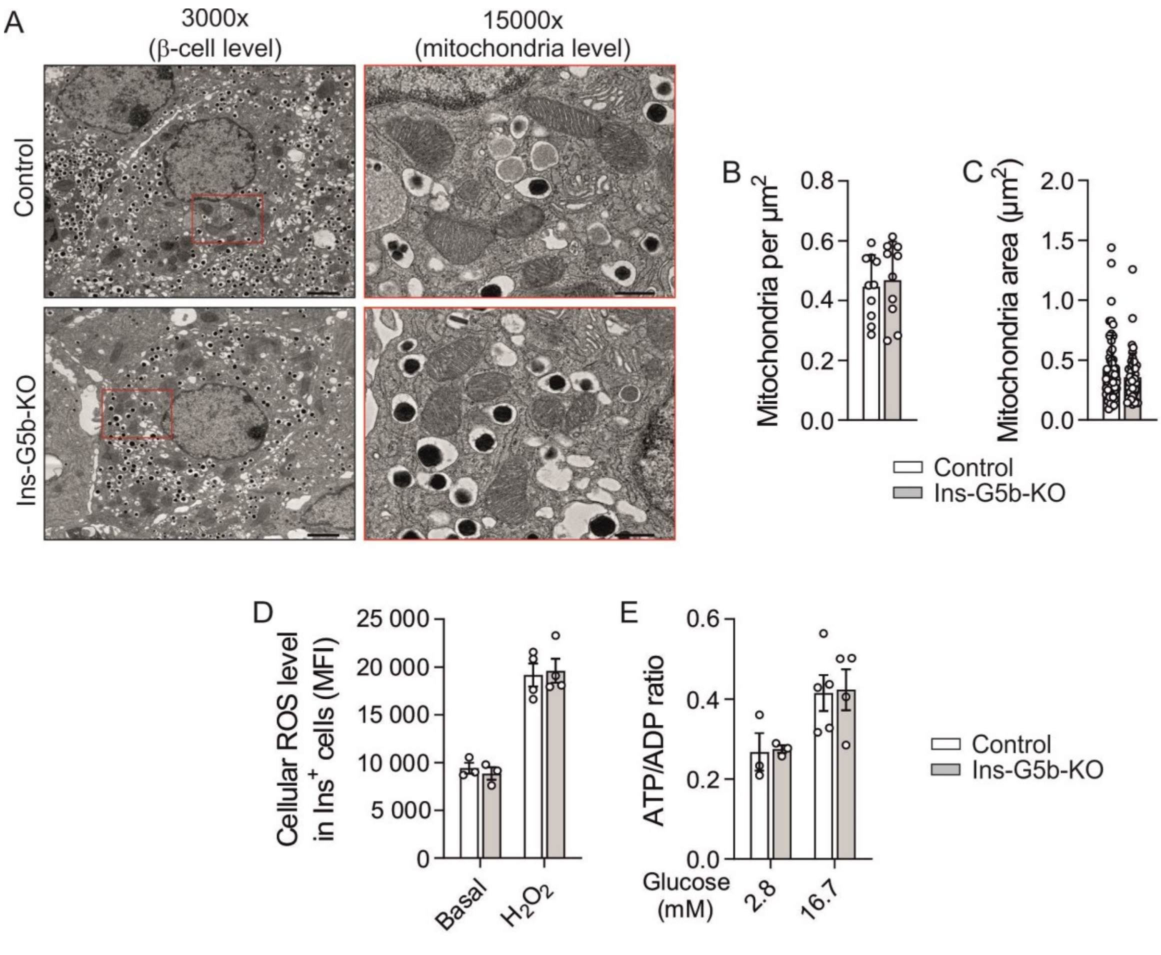

Supplementary Figure 3: A-C, Transmission electron microscopic characterization of mitochondria in primary mouse β-cells from HFD-fed mice: Exemplary photomicrographs (A) and statistical evaluation of mitochondria number (B) and size (C). **D,** ROS production (determined as deep red fluorescence intensity) was determined by flow cytometry in insulin-positive (Ins^+) cells freshly isolated from HFD-fed control and Ins-G5b-KOs. MFI indicates mean fluorescence intensity of the ROS sensor. **E,** ATP/ADP ratio in islets from HFD-fed controls and Ins-G5b-KOs. Islets were exposed to either 2.8 mM or 16.7 mM glucose.

Data are mean ± SEM; comparisons between groups were performed using unpaired, two-sided t test (B,C) or two-way ANOVA with Sidak's multiple comparisons (D,E).

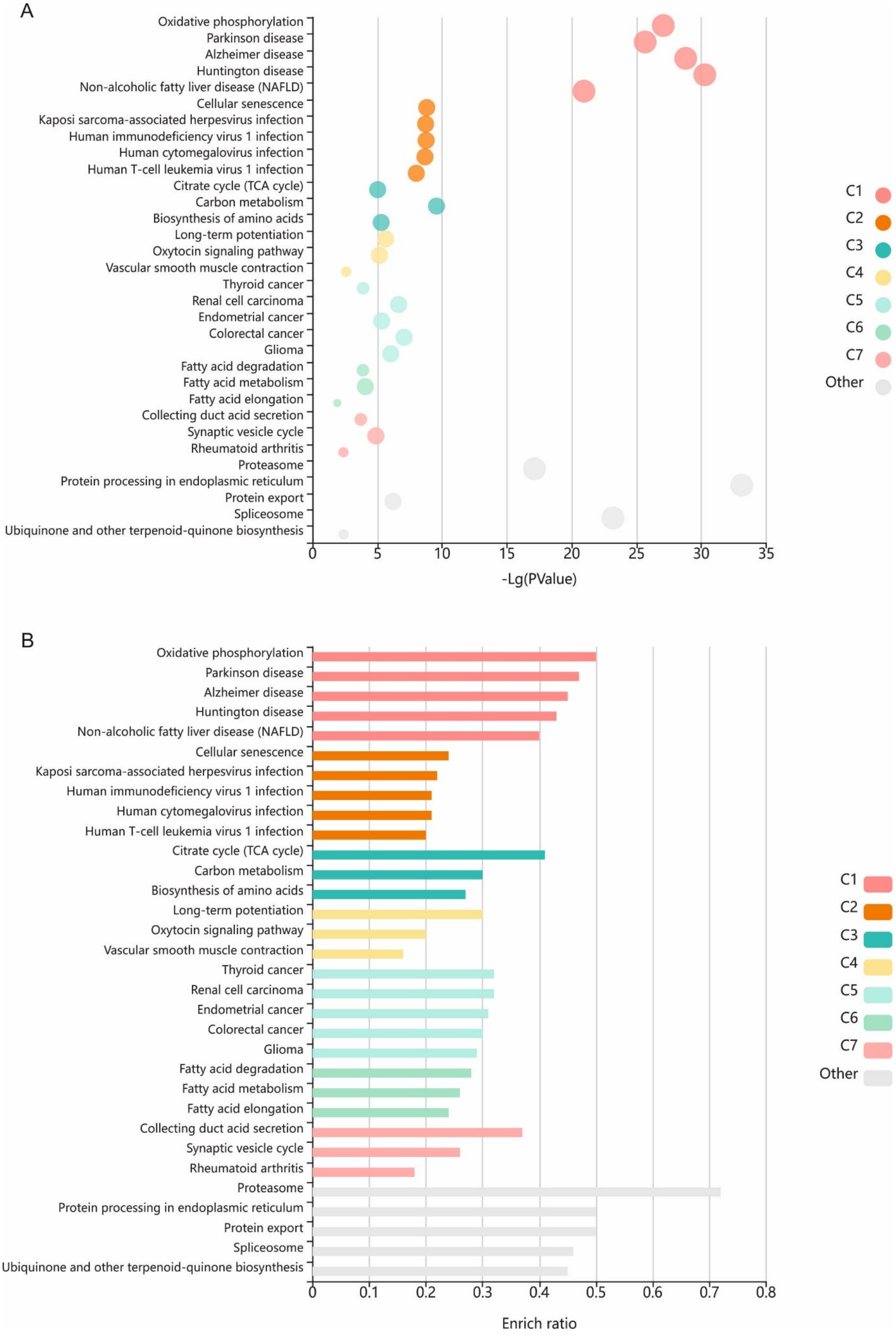
A
Oxidative phosphorylation
Parkinson disease
Alzheimer disease
Huntington disease
Non-alcoholic fatty liver disease (NAFLD)
Cellular senescence
Kaposi sarcoma-associated herpesvirus infection
Human immunodeficiency virus 1 infection
Human cytomegalovirus infection
Human T-cell leukemia virus 1 infection
Citrate cycle (TCA cycle)
Carbon metabolism
Biosynthesis of amino acids
Long-term potentiation
Oxytocin signaling pathway
Vascular smooth muscle contraction
Thyroid cancer
Renal cell carcinoma
Endometrial cancer
Colorectal cancer
Glioma
Fatty acid degradation
Fatty acid metabolism
Fatty acid elongation
Collecting duct acid secretion
Synaptic vesicle cycle
Rheumatoid arthritis
Proteasome
Protein processing in endoplasmic reticulum
Protein export
Spliceosome
Ubiquinone and other terpenoid-quinone biosynthesis
0 5 10 15 20 25 30 35
-Lg(PValue)
C1 C2 C3 C4 C5 C6 C7 Other
B
Oxidative phosphorylation
Parkinson disease
Alzheimer disease
Huntington disease
Non-alcoholic fatty liver disease (NAFLD)
Cellular senescence
Kaposi sarcoma-associated herpesvirus infection
Human immunodeficiency virus 1 infection
Human cytomegalovirus infection
Human T-cell leukemia virus 1 infection
Citrate cycle (TCA cycle)
Carbon metabolism
Biosynthesis of amino acids
Long-term potentiation
Oxytocin signaling pathway
Vascular smooth muscle contraction
Thyroid cancer
Renal cell carcinoma
Endometrial cancer
Colorectal cancer
Glioma
Fatty acid degradation
Fatty acid metabolism
Fatty acid elongation
Collecting duct acid secretion
Synaptic vesicle cycle
Rheumatoid arthritis
Proteasome
Protein processing in endoplasmic reticulum
Protein export
Spliceosome
Ubiquinone and other terpenoid-quinone biosynthesis
0 0.1 0.2 0.3 0.4 0.5 0.6 0.7 0.8
Enrich ratio
C1 C2 C3 C4 C5 C6 C7 Other

C

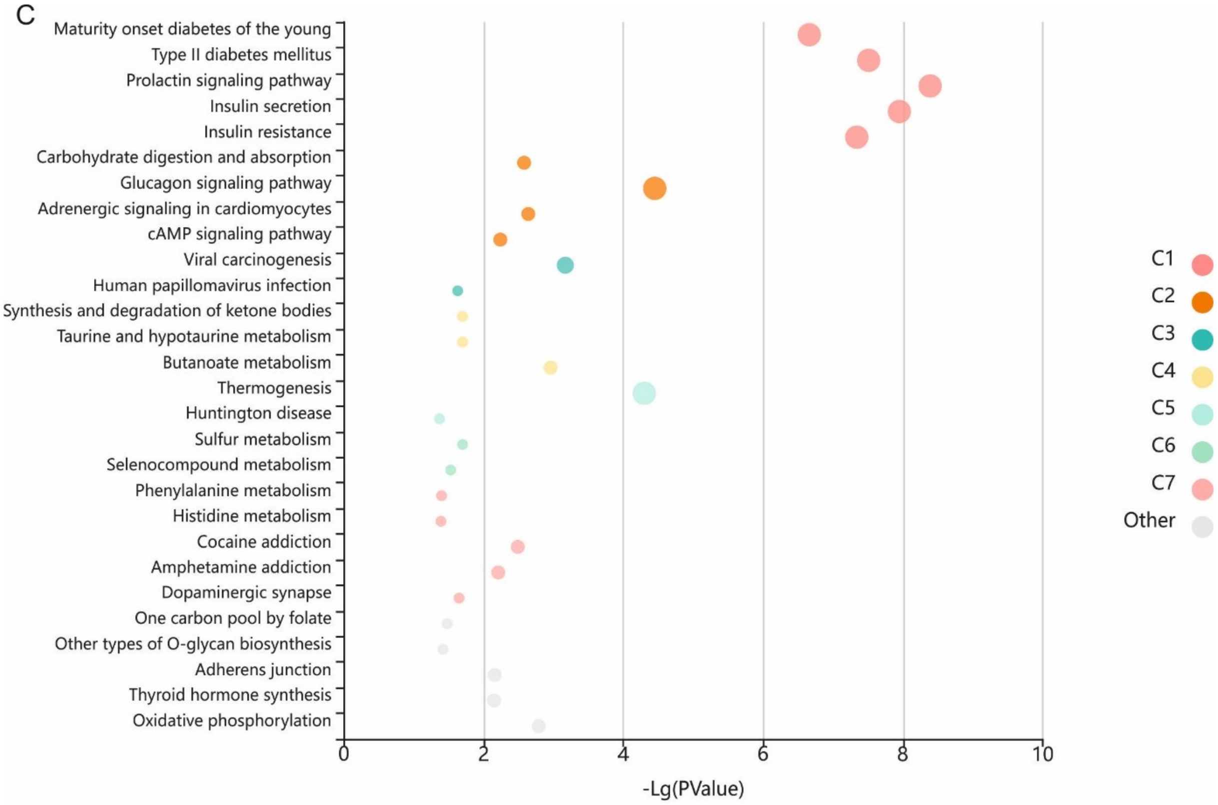
Maturity onset diabetes of the young
Type II diabetes mellitus
Prolactin signaling pathway
Insulin secretion
Insulin resistance
Carbohydrate digestion and absorption
Glucagon signaling pathway
Adrenergic signaling in cardiomyocytes
cAMP signaling pathway
Viral carcinogenesis
Human papillomavirus infection
Synthesis and degradation of ketone bodies
Taurine and hypotaurine metabolism
Butanoate metabolism
Thermogenesis
Huntington disease
Sulfur metabolism
Selenocompound metabolism
Phenylalanine metabolism
Histidine metabolism
Cocaine addiction
Amphetamine addiction
Dopaminergic synapse
One carbon pool by folate
Other types of O-glycan biosynthesis
Adherens junction
Thyroid hormone synthesis
Oxidative phosphorylation
0
2
4
6
8
10
-Lg(PValue)
C1
C2
C3
C4
C5
C6
C7
Other

D

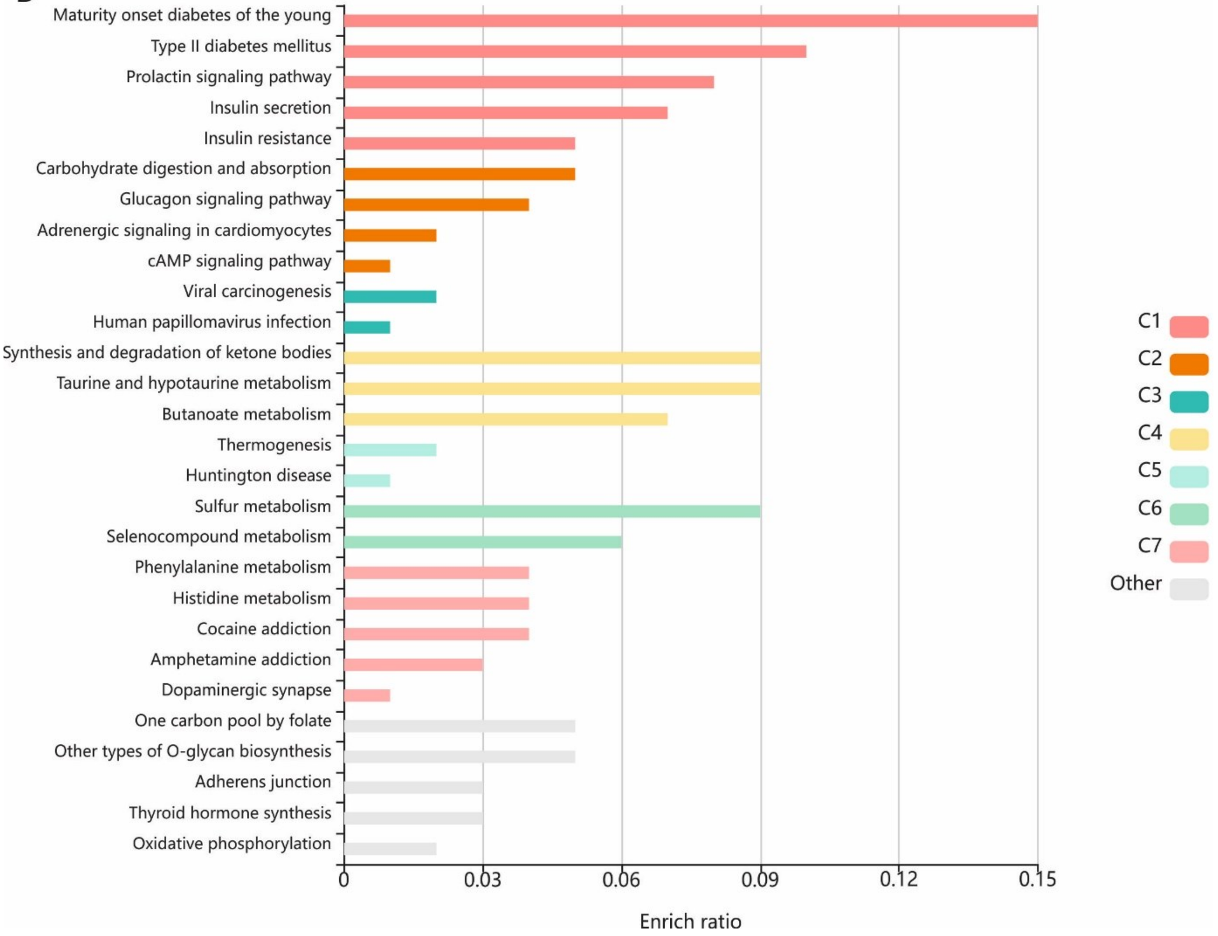
Maturity onset diabetes of the young
Type II diabetes mellitus
Prolactin signaling pathway
Insulin secretion
Insulin resistance
Carbohydrate digestion and absorption
Glucagon signaling pathway
Adrenergic signaling in cardiomyocytes
cAMP signaling pathway
Viral carcinogenesis
Human papillomavirus infection
Synthesis and degradation of ketone bodies
Taurine and hypotaurine metabolism
Butanoate metabolism
Thermogenesis
Huntington disease
Sulfur metabolism
Selenocompound metabolism
Phenylalanine metabolism
Histidine metabolism
Cocaine addiction
Amphetamine addiction
Dopaminergic synapse
One carbon pool by folate
Other types of O-glycan biosynthesis
Adherens junction
Thyroid hormone synthesis
Oxidative phosphorylation
0
0.03
0.06
0.09
0.12
0.15
Enrich ratio
C1
C2
C3
C4
C5
C6
C7
Other

Supplementary Figure 4: A-D, KEGG pathway enrichment analysis of upregulated differentially expressed genes (A, bubble chart with log-p value; B, bar chart with enrich ratio) and downregulated differentially expressed genes (C, bubble chart with log-p value; D, bar chart with enrich ratio) in GPRC5B-deficient β-cells. Data were analysed using KOBAS-I [1] platform for genes with absolute log2 fold change values of ≥0.04. “C1-7/other” represent different KEGG pathway clusters. For bubble charts (A,C), the node size represents 6 levels of enriched p-values (from small to large): [0.05,1], [0.01,0.05), [0.001,0.01), [0.0001,0.001), [1e-10,0.0001), [0,1e-10); In the bar charts (B,D), the length of the bar represents the enrich ratio, which is calculated as "input gene number"/ "background gene number".

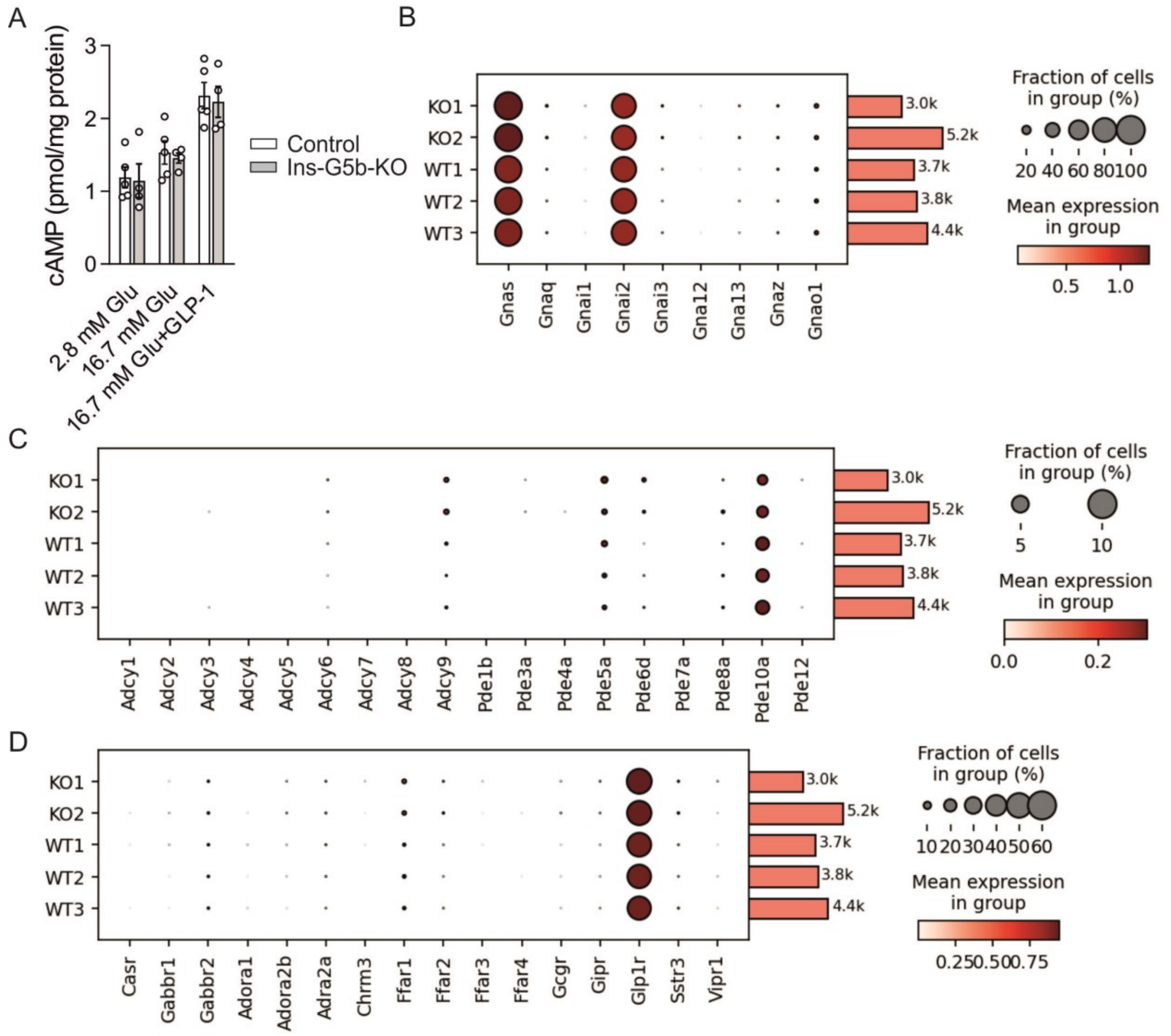

Supplementary Figure 5: A, Acute cAMP production in islets isolated from lean mice was measured following an overnight culture. After a 60-minute starvation period in 2.8 mM glucose, the islets were exposed to complete RPMI-1640 media containing either 2.8 mM glucose, 16.7 mM glucose, or 16.7 mM glucose with 100 nM GLP-1 for 30 minutes (n=5/4 mice). **B-D,** Effect of GPRC5B deficiency on the expression of heterotrimeric G-proteins (B), adenylyl cyclase and phosphodiesterase (C), and GPCRs (D) in β-cells of high fat diet-fed control mice (WT) and Ins-G5b-KOs (KO).

Data are means ± SEM; comparisons between groups were performed using two-way ANOVA with Sidak’s multiple comparisons (A).

Supplemental References

1. Bu D, Luo H, Huo P, Wang Z, Zhang S, He Z, Wu Y, Zhao L, Liu J, Guo J, Fang S, Cao W, Yi L, Zhao Y and Kong L. KOBAS-i: intelligent prioritization and exploratory visualization of biological functions for gene enrichment analysis. *Nucleic Acids Res*. 2021;49:W317-W325.
