# Supplementary material for: GPRC5B preserves a mature β cell state in obesity by controlling MafA expression
Source: JCI Insight. 2025 Sep 4;10(20):e194115. doi: 10.1172/jci.insight.194115 (PMC12581666; doi:10.1172/jci.insight.194115)

Uncropped blots used in manuscript

Fig 5A

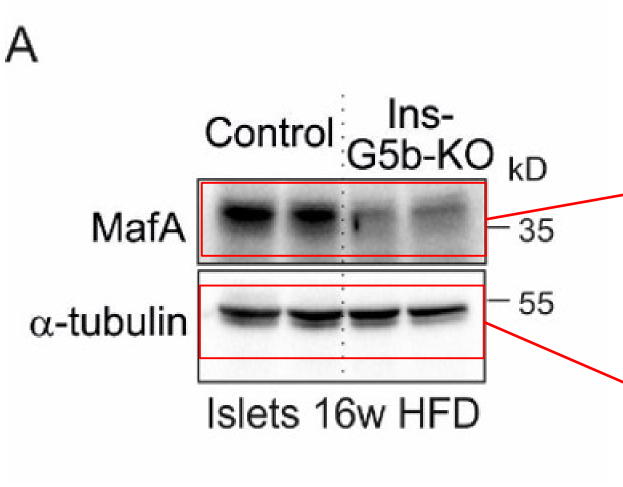

Full unedited gel for Figure 5A

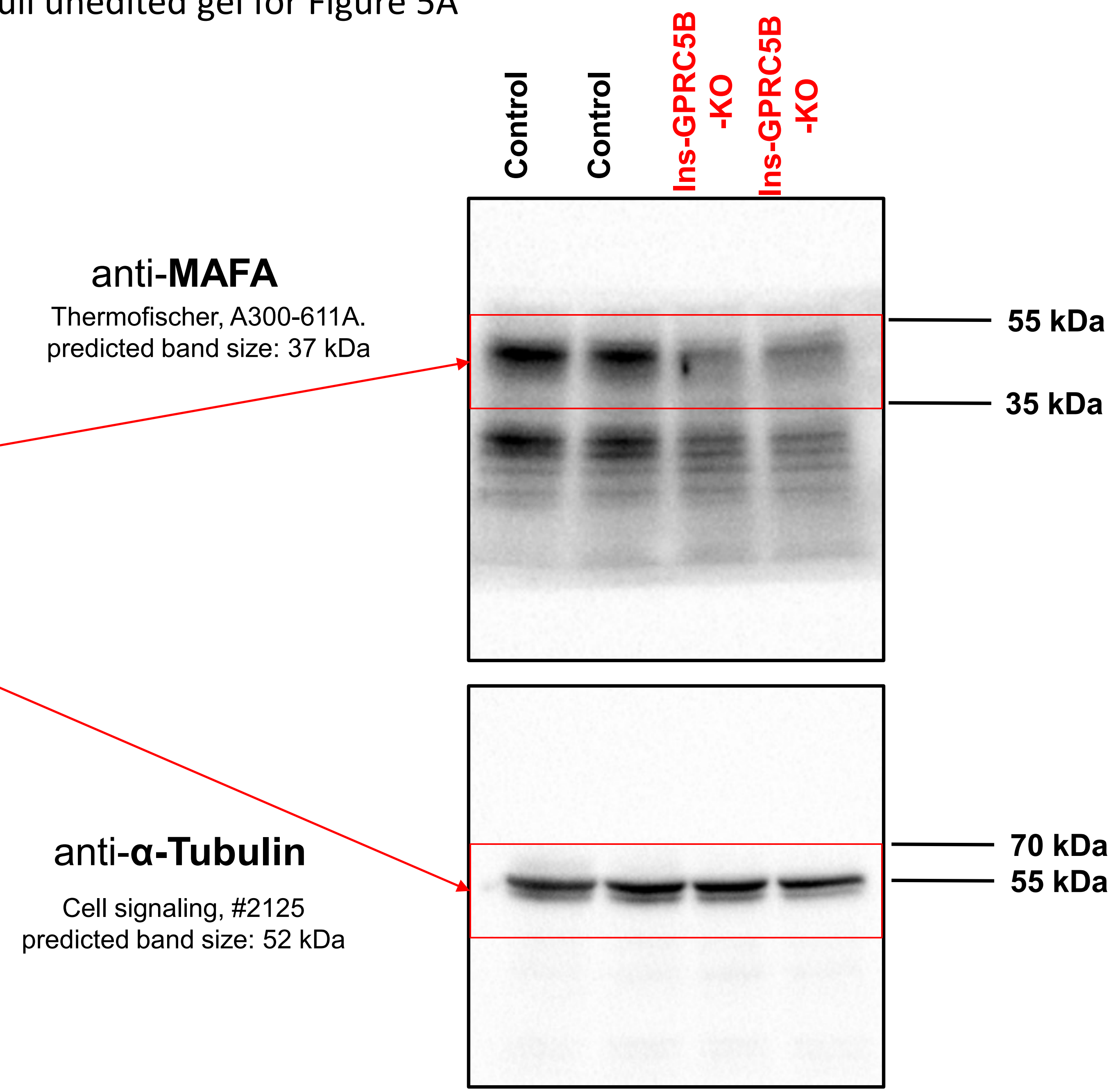

Fig 5D

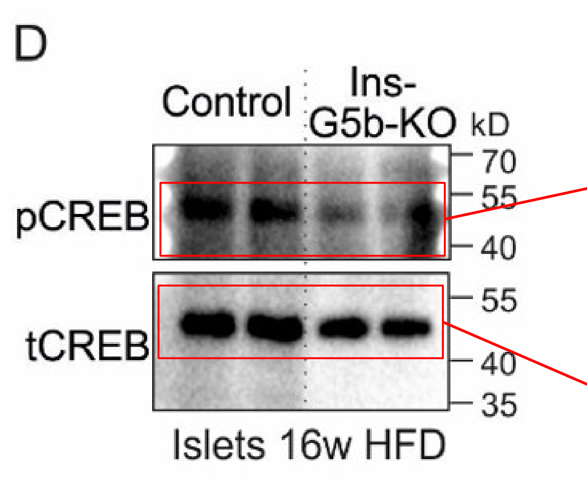

Full unedited gel for Figure 5D

anti-**pCREB (Ser133)**

Cell signaling, #9198s
predicted band size: 43 kDa

anti-**CREB**

Cell signaling, #9197
predicted band size: 43 kDa

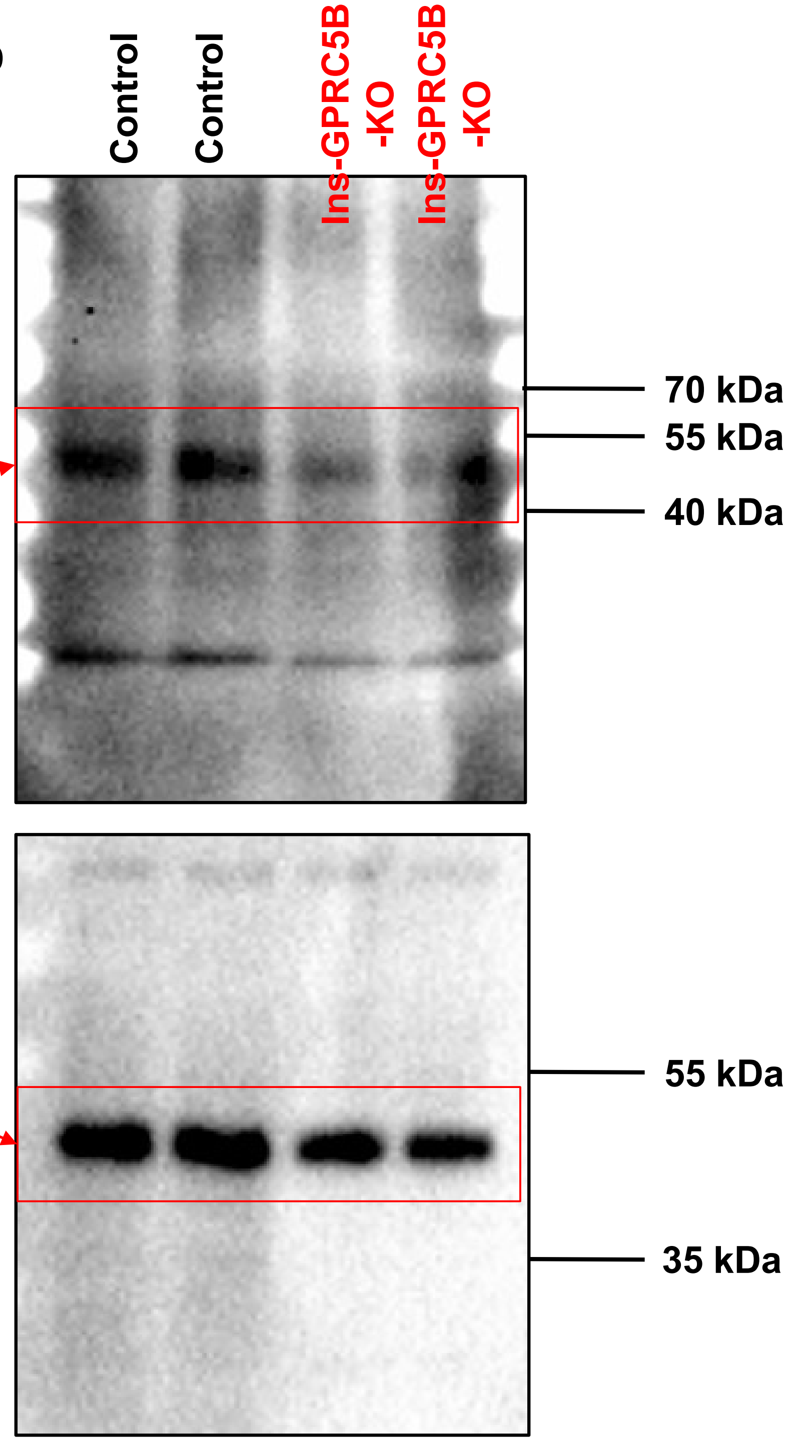

Fig 5F

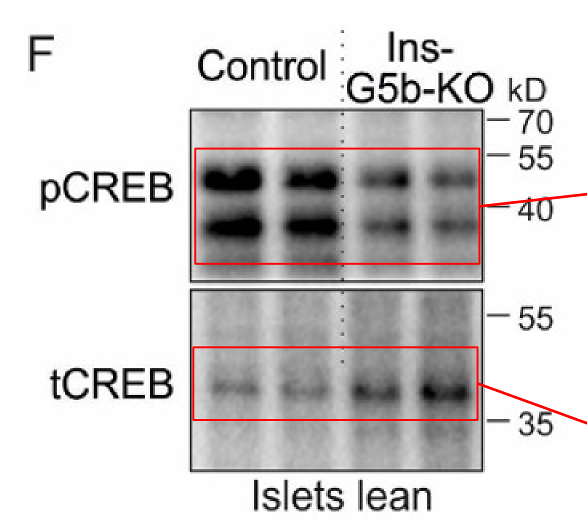

Full unedited gel for Figure 5F

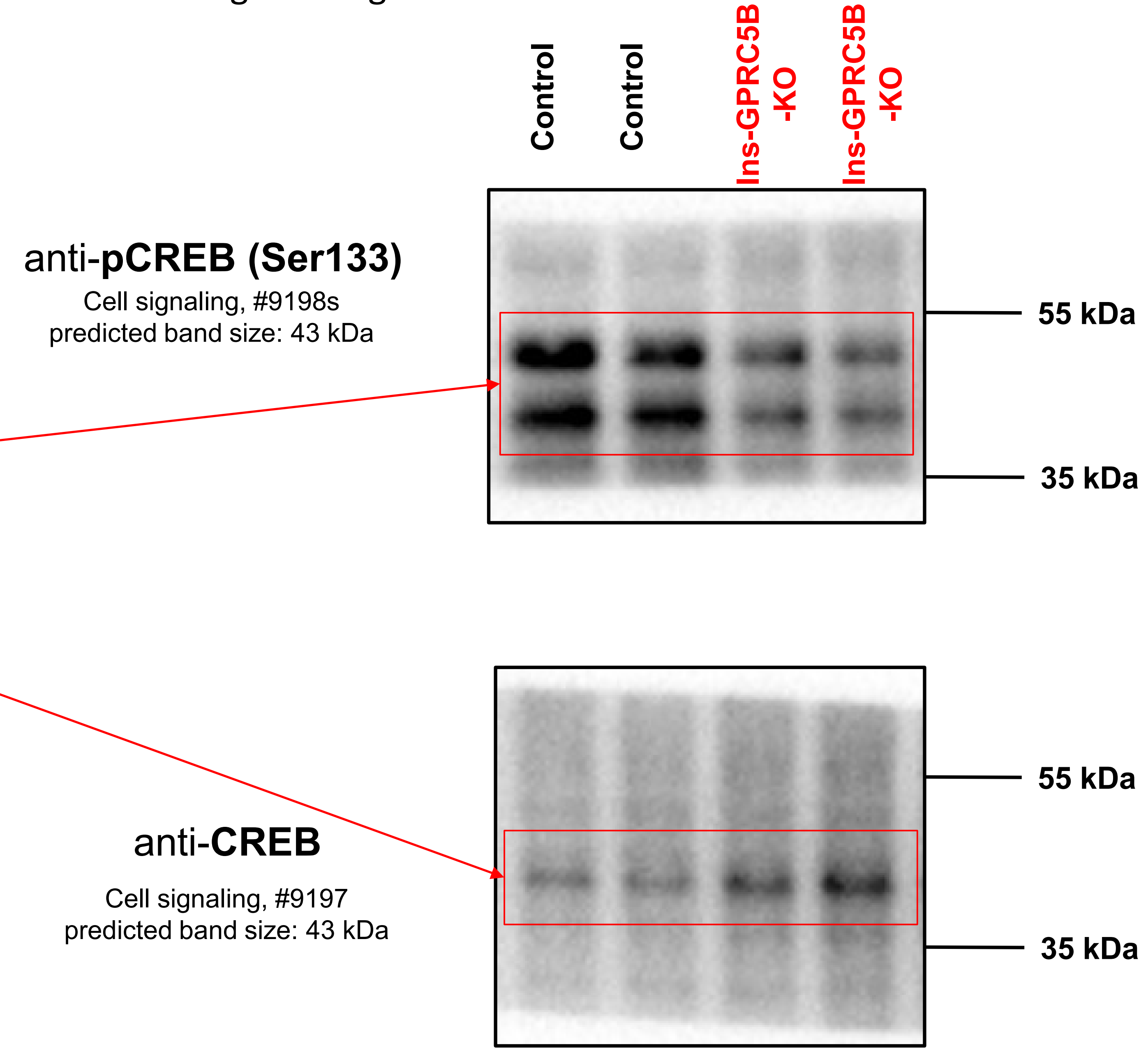

Fig 5H

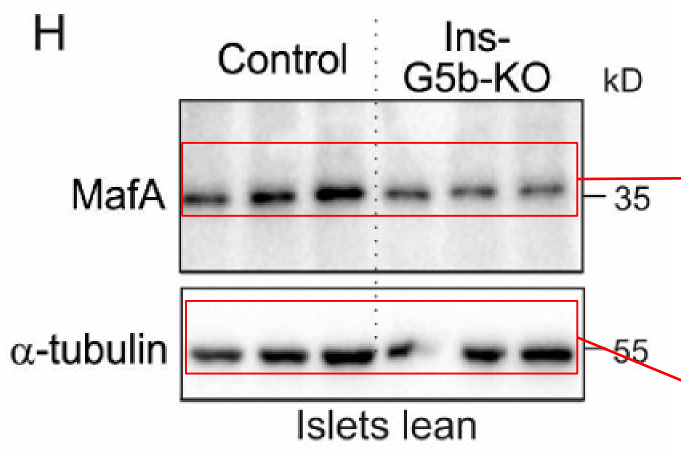

Full unedited gel for Figure 5H

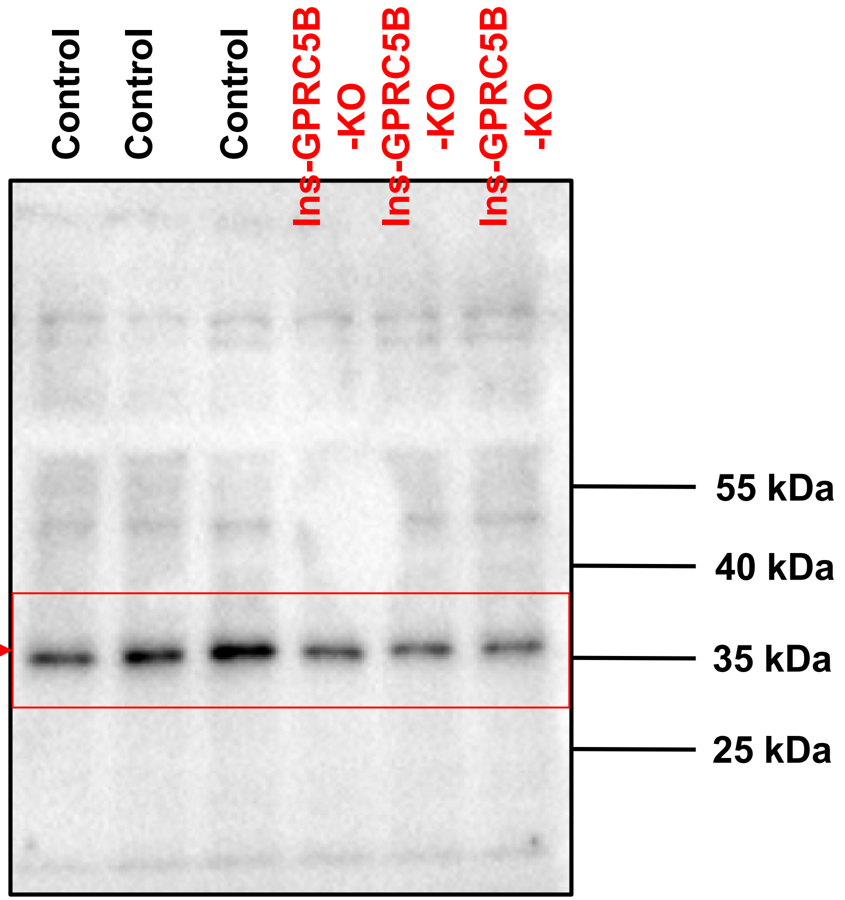

anti-**MAFA**

Thermofischer, A300-611A.
predicted band size: 37 kDa

anti-**α-Tubulin**

Cell signaling, #2125
predicted band size: 52 kDa

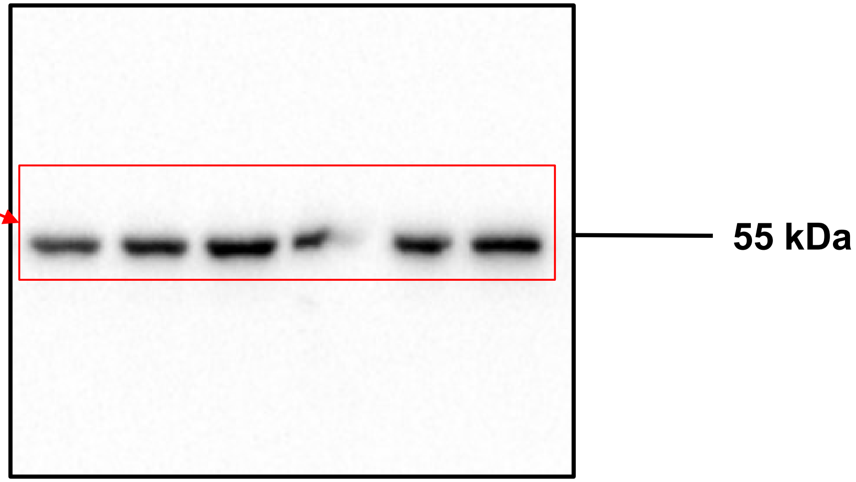

Supplement: Unedited blot and gel images [file jciinsight-10-194115-s153.pdf]
